# Identification of Environmental Predictors and Their Spatial Heterogeneity for VMS-Derived Fishing Duration in the Argentine Shortfin Squid Fishery Across the Southwest Atlantic Ocean

**DOI:** 10.3390/biology15191735

**Published:** 2026-10-01

**Authors:** Famou Zhang, Kangbo Li, Ming Gao, Chen Liu, Chenshuai Dong, Chen Li, Shuaiwen Zhang, Heng Zhang

**Affiliations:** 1 Key Laboratory of Oceanic and Polar Fisheries, Ministry of Agriculture and Rural Affairs, East China Sea Fisheries Research Institute, Chinese Academy of Fishery Sciences, Shanghai 200090, China; 2 College of Marine Sciences, Shanghai Ocean University, Shanghai 201306, China; 3 School of ecology and environment, Anhui Normal University, Wuhu 241002, China; 4 Zhejiang Intertion Information Technology Co., Ltd., Zhoushan 316100, China; 5 Wenchang Innovation Research Center of East China Sea Fisheries Research Institute, Chinese Academy of Fishery Sciences, Wenchang 571343, China

**Keywords:** *Illex argentinus*, vessel monitoring system, fishing duration, environmental predictors, spatial heterogeneity, SHapley Additive exPlanations

## Abstract

Argentine shortfin squid (*Illex argentinus*) fishing grounds in the Southwest Atlantic extend across the Argentine Exclusive Economic Zone (EEZ) and adjacent high seas, where environmental contrasts may lead to spatial heterogeneity. In this study, Vessel Monitoring System data from December 2020 to June 2025 were used to derive fishing duration as an indicator of fishing-ground use intensity. Conventional and derived environmental variables described vertical temperature and salinity differences, surface gradients, and topography. K-means clustering identified fishing-ground units, while CatBoost, SHapley Additive exPlanations, and accumulated local effects identified major environmental predictors and high-response ranges. No persistent fishing-ground center was identified within the EEZ, whereas the high seas were divided at 44.27° S into northern and southern fishing grounds with persistent centers. Seabed slope, bathymetry, and sea surface temperature were important predictors across the full area and subregions, but their rankings and high-response ranges differed among regions. Under LOSO validation, the full-study-area model yielded R^2^ = 0.083 ± 0.104 and Spearman’s ρ = 0.4485 ± 0.0903, indicating limited absolute predictive performance but some relative ranking capability. Modeling the full area as a single unit obscures regional differences in predictor combinations and response ranges. The results demonstrate that region-specific modeling can characterize spatially varying environmental associations and support differentiated management.

## Simple Summary

The Argentine shortfin squid (*Illex argentinus*) is an important fishery resource in the Southwest Atlantic, and its fishing grounds extend across the Argentine Exclusive Economic Zone and adjacent high seas. Environmental conditions, including water temperature, salinity, water-column structure, and seabed topography, differ among fishing areas, and their associations with fishing-ground use may vary spatially. In this study, fishing duration was derived from Vessel Monitoring System data collected between December 2020 and June 2025 to serve as an indicator of fishing-ground use intensity. Conventional oceanographic data were combined with derived variables describing temperature and salinity differences between depth layers, spatial changes in surface conditions, and seabed features. No long-term fixed fishing-ground center was found within the Exclusive Economic Zone, whereas the high seas contained two relatively stable fishing grounds in the north and south. Derived variables, including seabed slope, were important across all regions, whereas the associations of vertical temperature and salinity differences, environmental gradients, and distance from the shelf break with fishing-ground use varied among regions. These findings indicate that fishing duration reflects how vessels use fishing grounds, highlighting that regional analyses can improve monitoring of fishing-ground use and support area-specific fishery management.

## 1. Introduction

The Argentine shortfin squid (*Illex argentinus*) is the most important commercial cephalopod in the Southwest Atlantic. It is widely distributed across the Patagonian Shelf, the shelf break, and the adjacent high seas, where areas featuring high resource aggregation develop into highly productive fishing grounds. It is one of the principal target species of jigging and trawl fisheries both within the Argentine Exclusive Economic Zone (EEZ) and near adjacent high seas [1,2,3]. The species has a short life cycle, rapid growth, and pronounced interannual variability in abundance, and its recruitment, migration, and fishing-ground distribution are highly sensitive to changes in the marine environment [4]. The population structure of *Illex argentinus* is complex, with different stocks varying in spawning season and in the distributions of their spawning and feeding grounds. Among these stocks, the South Patagonian Stock (SPS) is a major target of fisheries in the Southwest Atlantic. Its life history includes hatching over the outer shelf, the movement of paralarvae and immature individuals toward feeding grounds on the Patagonian Shelf, and the spawning migration of mature individuals along the shelf break [5]. In addition, the distribution of *Illex argentinus* and the locations of its fishing grounds vary seasonally and are jointly influenced by sea surface temperature (SST), water-mass structure, frontal activity, and prey availability [6,7]. Therefore, identifying environmental predictors associated with changes in VMS-derived fishing-ground use intensity can help characterize high-use fishing grounds and their regional variations, while also providing an interpretable environmental basis for dynamic fishing-ground monitoring, fishing-area identification, and management.

Catch and catch per unit effort (CPUE) are commonly used to represent the abundance or exploitable resource level of *Illex argentinus* because these metrics are derived directly from fishing logbooks, facilitating comparisons of resource variation among years, months, and regions [1,6]. Approaches based on fishing-location distributions and fishing-ground classifications convert discrete fishing records into fishing hotspots or graded fishing grounds, thereby reflecting vessel selection among different waters and the spatial aggregation of fishing effort [8,9]. The habitat suitability index (HSI) further integrates multiple environmental conditions into spatially continuous estimates of habitat suitability and can be used to evaluate seasonal and environmentally driven shifts in potential fishing grounds [10]. However, catch and CPUE can be affected by vessel performance, fishing strategies, and manual recording errors in fishing logbooks. Fishing locations and graded fishing grounds generally indicate only whether vessels operated at a given location and therefore cannot readily distinguish short-duration operations from prolonged, continuous fishing activity [8]. By contrast, vessel-position data continuously record vessel location, time, speed, and course, providing information at a higher spatiotemporal resolution for extracting fishing effort from actual fishing operations [11]. Although fishing duration derived from continuous fishing trajectories cannot directly represent resource abundance, it reflects the degree of sustained fishing activity within a defined spatiotemporal domain. It can therefore serve as a process-based indicator of fishing-ground use intensity and provide a continuous measure for examining spatial variation in fishing fleet activity. Previous studies have used Automatic Identification System vessel-position data to classify trawl fishing grounds in the Southwest Atlantic high seas into different levels and to analyze and predict their spatiotemporal variation [8,9]. Vessel Monitoring System (VMS) data have also been used to identify the operating states of squid-jigging vessels in the Eastern Pacific and to estimate fishing duration [12]. However, few studies have used VMS-derived fishing duration as an indicator of fishing-ground use intensity to analyze how environmental conditions affect the Argentine shortfin squid jigging fishery in the Southwest Atlantic.

SST, chlorophyll-a concentration (Chl-a), sea surface height (SSH), mixed layer depth (MLD), and bathymetry are commonly used oceanographic variables in analyses of the resource abundance and fishing-ground locations of *Illex argentinus* [6,9]. These variables describe the basic environmental conditions of fishing grounds. However, *Illex argentinus* exhibits pronounced horizontal migration and diel vertical movement, and its spatial distribution is therefore not influenced solely by environmental conditions at the sea surface or within a single depth layer. The 50 m layer may represent an important depth interface associated with abundance variation near the Patagonian Shelf, while temperature variation within the 50–100 m layer is also closely related to its spatial distribution [13]. In studies of the purpleback flying squid in the Northwest Indian Ocean, temperature differences between depth layers have been used to represent the vertical thermal structure and have shown nonlinear relationships with CPUE [14]. In addition to vertical temperature differences, vertical salinity differences do not directly reflect thermal conditions but can indicate differences in water masses and density structure among depth layers. This is relevant because the migration of *Illex argentinus* between the shelf and slope is closely associated with the seawater density structure and water-mass movement pathways [5]. Furthermore, pronounced thermal fronts, water-mass exchange, and productivity variations occur near the Patagonian shelf break, and fishing activity is often concentrated along fronts and adjacent environmental transition zones [15,16,17]. Therefore, environmental values from a single depth layer or grid cell describe only local environmental conditions and cannot simultaneously characterize the vertical water-column structure, environmental variation in adjacent waters, and the topographic transition from the shelf to the slope. Constructing derived variables representing vertical temperature and salinity differences, horizontal environmental gradients, and topographic transitions can provide a more comprehensive characterization of the environmental conditions associated with fishing grounds of high use intensity and help identify major environmental predictors associated with fishing-ground use intensity in the *Illex argentinus* fishery.

The distribution of *Illex argentinus* and the extent of its fishing grounds span the Argentine EEZ and the adjacent high seas, forming a typical transboundary fishery system [18]. The fishing locations of the high-seas trawl fishery in the Southwest Atlantic are not uniformly distributed. During the early fishing season, from December to April of the following year, fishing operations are concentrated mainly in the southern, higher-latitude fishing grounds, whereas northern fishing grounds at lower latitudes gradually emerge during the middle and later stages of the fishing season, from March to June [8,10]. Although previous studies have described such spatial and seasonal variations in fishing-ground distribution, it remains unclear whether environmental predictors and their response patterns associated with fishing-ground use intensity are consistent among spatially distinct fishing-ground units. This spatial differentiation suggests that the associations between environmental conditions and fishing-ground use vary among fishing-ground units. Applying a single model across the entire study area may mask region-specific predictive associations and response ranges, thereby limiting the characterization of spatial heterogeneity. Therefore, regional modeling was used to test whether environmental predictors and their response relationships differed among distinct fishing-ground units.

To further investigate the relationship between fishing-ground use intensity and the marine environment in the *Illex argentinus* jigging fishery in the Southwest Atlantic, and to examine spatial variation in these model-derived environmental associations, the following three objectives were addressed: (1) to identify the fishing states of squid-jigging vessels from continuous VMS trajectories in waters within the Argentine EEZ and in the Southwest Atlantic high seas, extract fishing duration as the response variable to characterize fleet-level fishing-ground use intensity, and assess its spatial variation; (2) to introduce derived variables, including vertical temperature and salinity differences, horizontal gradients of surface environmental variables, seabed slope, and distance to the shelf break, in addition to commonly used surface and subsurface oceanographic variables, and to evaluate their complementary value in characterizing the vertical water-column structure, environmental transitions, and topographic settings, thereby enabling a more comprehensive identification of model-derived associations between fishing-ground use intensity and the marine environment; and (3) to assess the presence of relatively stable fishing grounds based on monthly fishing-ground centers and their spatial characteristics, develop models for the full study area and individual subregions, compare the predictive importance and response characteristics of major environmental predictors, and assess spatial variation in their associations with fishing-ground use intensity. These analyses aim to provide a scientific basis for the regional monitoring and differentiated management of *Illex argentinus* fishing grounds in the Southwest Atlantic.

## 2. Materials and Methods

### 2.1. Data Sources

#### 2.1.1. Vessel Position Data

**Figure 1:**
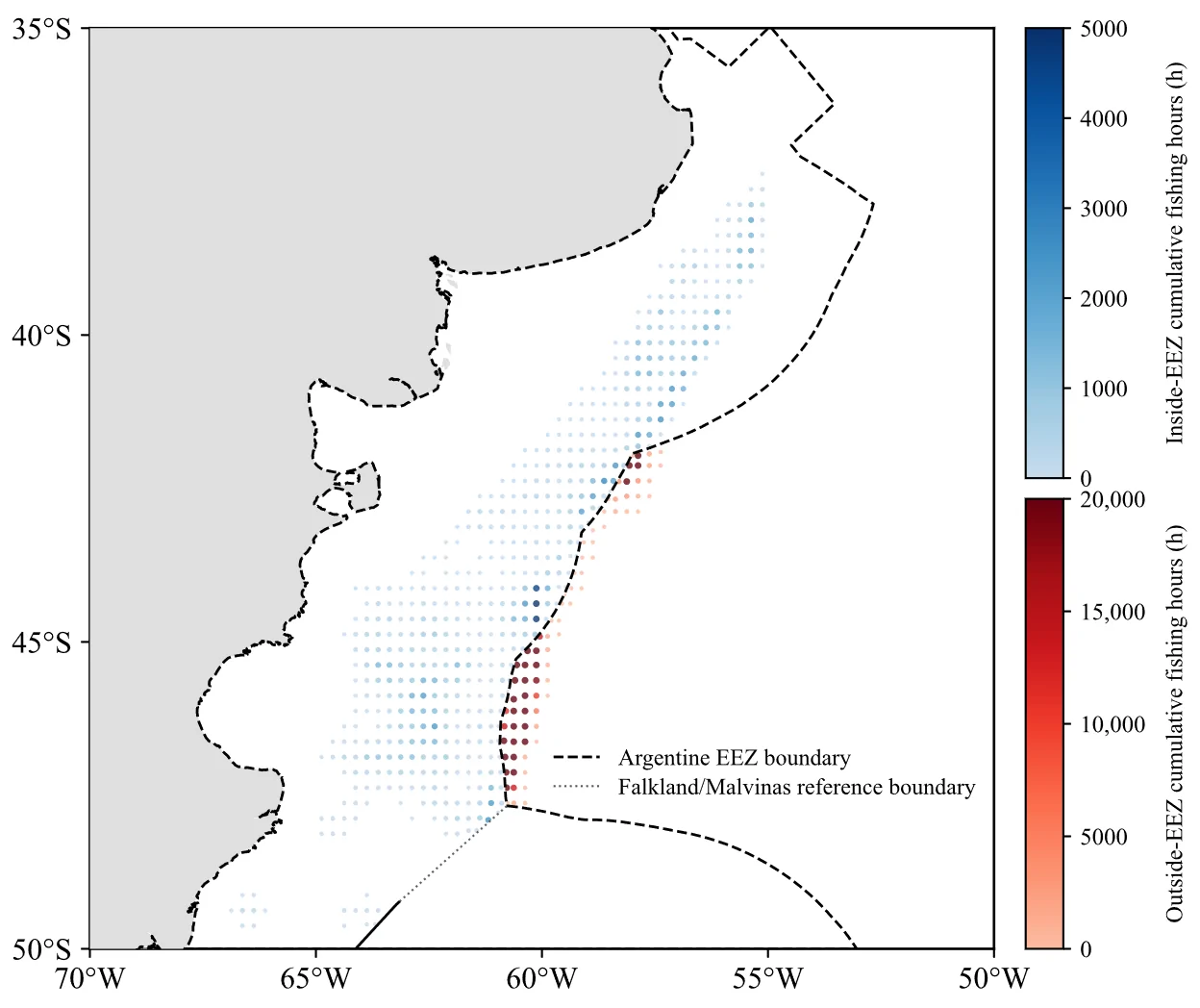
Spatial distribution of cumulative fishing hours for Argentine shortfin squid-jigging vessels in the Southwest Atlantic from December 2020 to June 2025.

VMS data were obtained for Chinese squid-jigging vessels operating in the *Illex argentinus* fishing grounds in the Southwest Atlantic from December 2020 to June 2025. Based on the seasonal characteristics of the *Illex argentinus* jigging fishery, the period from 1 December of each year to 30 June of the following year was defined as one fishing season. Each fishing season was named according to its ending year, resulting in five fishing seasons. The study area included fishing grounds within the Argentine EEZ and in the adjacent high seas outside the EEZ (Figure 1). Fishing operations within and outside the EEZ differ in terms of access authorization, operational management, and fleet composition. Moreover, vessels operating within and outside the EEZ generally do not shift between the two areas during the same fishing season. The two fleets were therefore treated as relatively independent operational groups [19]. Approximately 220–250 vessels were included in each fishing season, and the ratio of squid-jigging vessels operating within the EEZ to those operating outside the EEZ was approximately 1:7. The VMS records included vessel name, timestamp, longitude, latitude, vessel speed in knots (kn), and course in degrees, with a position-reporting frequency of approximately one record per hour. These data captured the spatiotemporal activity of squid-jigging vessels throughout each fishing season and provided the basis for the subsequent identification of fishing states and extraction of fishing duration.

#### 2.1.2. Marine Environmental Data

*Illex argentinus* exhibits migratory behavior and pronounced diel vertical movement [20], and the distribution of its fishing grounds is closely associated with oceanographic conditions such as temperature, salinity, ocean currents, sea surface height, local environmental gradients, and primary productivity [21]. Accordingly, daily oceanographic variables covering the same period as the VMS data, from December 2020 to June 2025, were selected. These variables included SST, sea surface salinity (SSS), SSH, MLD, surface current speed, chlorophyll-a concentration (Chl-a), temperature and salinity at approximately 50 m (T50 and S50), temperature and salinity at approximately 100 m (T100 and S100), bottom temperature, and bathymetry. Except for Chl-a, which had an original spatial resolution of 0.25°, all oceanographic variables had an original spatial resolution of 0.083°. The data were obtained primarily from the Copernicus Marine Data Store (https://data.marine.copernicus.eu/products (accessed on 30 July 2026)), including the Global Ocean Physics Multiyear and Global Ocean Biogeochemistry Multiyear products.

Some environmental variables required further processing from the original products. Surface current speed was calculated from the eastward and northward current-velocity components to characterize current-field intensity. The temperature and salinity variables representing the approximately 50 m and 100 m layers did not correspond directly to individual standard depth levels. Instead, T50 and S50 were derived from the two adjacent depth levels at 47.4 and 55.8 m, whereas T100 and S100 were derived from those at 92.3 and 109.7 m. Values at exactly 50 and 100 m were obtained via linear interpolation between the corresponding adjacent depth levels. When only one adjacent level contained a valid value, that value was retained, whereas values remained missing when both adjacent levels were unavailable. Surface wind data were additionally obtained from the Copernicus Climate Data Store, utilizing the “ERA5 hourly data on single levels” dataset (https://cds.climate.copernicus.eu/datasets (accessed on 30 July 2026)). These data had an hourly temporal resolution and a spatial resolution of 0.25° and were provided as 10 m eastward and northward wind components, from which the total wind speed was calculated. They were used only as auxiliary information for vessel-state classification and were not included as environmental predictors in the subsequent model analyses.

### 2.2. Data Processing

#### 2.2.1. Extraction of Fishing Duration

Before initiating fishing operations, squid-jigging vessels targeting *Illex argentinus* in the Southwest Atlantic deploy a parachute sea anchor and then conduct jigging operations while drifting with the currents. As continued drifting gradually carries a vessel away from its original fishing area, the sea anchor must be retrieved and the vessel must relocate to search for another suitable fishing ground. Because the daily operating locations of vessels change continuously through current-driven drifting and transit, continuous VMS position data can effectively reflect vessel operating processes and provide the basis for extracting fishing duration. Therefore, a two-component Gaussian mixture model (GMM) was fitted to the mixed vessel-speed distribution representing low-speed and transit states [22]. The speed at which the probability densities of the two Gaussian components were equal was used as the threshold for separating the two states, yielding a critical speed of approximately 1.9 kn. Surface wind speed, the duration of continuous low-speed movement, and a three-point moving-window method were subsequently incorporated to classify vessel activity into four states: fishing, drifting, transit, and weather avoidance [23]. However, point-level ground-truth labels for vessel operating states were unavailable. Therefore, some uncertainty may remain in the VMS-based fishing-state classification, and this uncertainty may propagate into the subsequently derived fishing duration.

After the vessel states had been classified, consecutive sequences of positions identified as fishing were used to extract fishing duration. For each pair of consecutive fishing positions from the same squid-jigging vessel, the time interval between the two positions was regarded as a valid fishing period. When a fishing period crossed a calendar date or spatial grid-cell boundary, it was partitioned according to the temporal and spatial relationships between the two positions and allocated to the corresponding dates and grid cells [24]. Fishing duration was then summed across all vessels within each standard 0.25° × 0.25° grid cell on each date to obtain grid-day fishing duration. This variable was subsequently matched with the oceanographic data at the same spatial and temporal scales and used as the response variable for identifying environmental factors and comparing regional differences.

#### 2.2.2. Fishing-Ground Regionalization

The cumulative fishing duration over the five fishing seasons showed varying degrees of spatial aggregation in waters inside and outside the EEZ, as well as between the northern and southern parts of the area outside the EEZ (Figure 1). To avoid masking regional differences in model-derived environmental associations by fitting a single model across the full study area, the study area was first divided into waters inside and outside the EEZ according to the EEZ boundary. Each area was then examined separately to determine whether persistent centers of fishing activity were present. Daily fishing-duration data were aggregated to the monthly scale. For each month, the 80th percentile of monthly cumulative fishing duration was calculated across grid cells with fishing records, and grid cells with values at or above this threshold were defined as fishing-ground hotspot cells for that month [25]. To evaluate the robustness of this operational hotspot definition, a sensitivity analysis was additionally conducted using the 40th, 50th, 60th, 70th, and 80th percentiles, while all subsequent centroid calculation and regionalization procedures were kept unchanged. A fishing duration-weighted centroid was then calculated from the hotspot cells to represent the location of the monthly fishing-ground center.

To determine whether persistent fishing-ground centers recurred across months and fishing seasons inside and outside the EEZ, K-means clustering was applied separately to the monthly fishing-ground centers in the two areas, with different numbers of clusters tested (k=2−5) [26]. For each value of k, clustering performance was evaluated using the silhouette score, Calinski–Harabasz index, Davies–Bouldin index, and within-cluster sum of squares (inertia). Regionalization was not determined by any single clustering index. Instead, the assessment considered whether the fishing-ground centers generated under different values of k were clearly separated in space, whether they recurred across multiple fishing seasons, and whether they were suitable as spatial units for subsequent separate modeling. If an area did not exhibit persistent and clearly separated long-term fishing-ground centers, it was retained as a single spatial unit; otherwise, subregional boundaries were delineated according to the locations of the identified centers.

#### 2.2.3. Construction and Matching of Derived Environmental Variables

After the major environmental variables had been integrated, additional derived variables were constructed to characterize the vertical structure, topographic constraints, and horizontal gradients. Major environmental variables with an original spatial resolution of 0.083° were aggregated to the 0.25° analysis grid using the mean of valid source cells, whereas Chl-a was retained at its native 0.25° resolution. No spatial interpolation was applied. All derived variables were harmonized to a 0.25° × 0.25° grid and matched with the fishing-duration data at a daily scale. Environmental conditions associated with *Illex argentinus* fishing grounds involve not only surface oceanographic conditions but also the upper-water-column thermohaline structure and subsurface conditions [27]. Based on SST, SSS, T50, S50, T100, and S100, temperature and salinity differences were calculated between the surface and 50 m, between 50 and 100 m, and between the surface and 100 m. This procedure produced six vertical-difference variables: $\Delta T_{0-50}$, $\Delta T_{50-100}$, $\Delta T_{0-100}$, $\Delta S_{0-50}$, $\Delta S_{50-100}$, and $\Delta S_{0-100}$.

The topographic-constraint variables comprised seabed slope and distance to the shelf break. Seabed slope was calculated from the static bathymetric field at a spatial resolution of 0.25° and represented the combined rate of change in seabed depth in the zonal and meridional directions, with the unit converted to m km^−1^. The 200 m isobath was used as an approximate reference for the shelf break [28], and the great-circle distance from the center of each grid cell to the center of the nearest grid cell along the 200 m isobath was calculated.

Horizontal-gradient variables were used to characterize the intensity of spatial variation in local environmental fields. The magnitudes of the horizontal gradients were calculated separately for SST, SSH, surface current speed, and Chl-a [29], as shown in Equation (1):

(1) $$G_{x}=\sqrt{{\left(\frac{\partial X}{\partial x}\right)}^{2}+{\left(\frac{\partial X}{\partial y}\right)}^{2}}$$

In Equation (1), $G_{x}$ denotes the horizontal-gradient magnitude of environmental variable X; X represents SST, SSH, surface current speed, or Chl-a; x and y denote the zonal and meridional spatial distances, respectively; and ∂X/∂x and ∂X/∂y represent the corresponding rates of spatial change in the zonal and meridional directions. For surface current speed, the scalar speed field was first calculated from the eastward and northward current components at the original 0.083° resolution. For SST, SSH, and surface current speed, which had an original spatial resolution of 0.083°, the gradients were first calculated at the original resolution and then aggregated to the 0.25° grid. For Chl-a, which had an original spatial resolution of 0.25°, the gradient was calculated directly at that resolution. These horizontal-gradient variables represented only the intensity of local spatial variation and were not directly equivalent to the locations of oceanic fronts in the strict sense.

**Figure 2:**
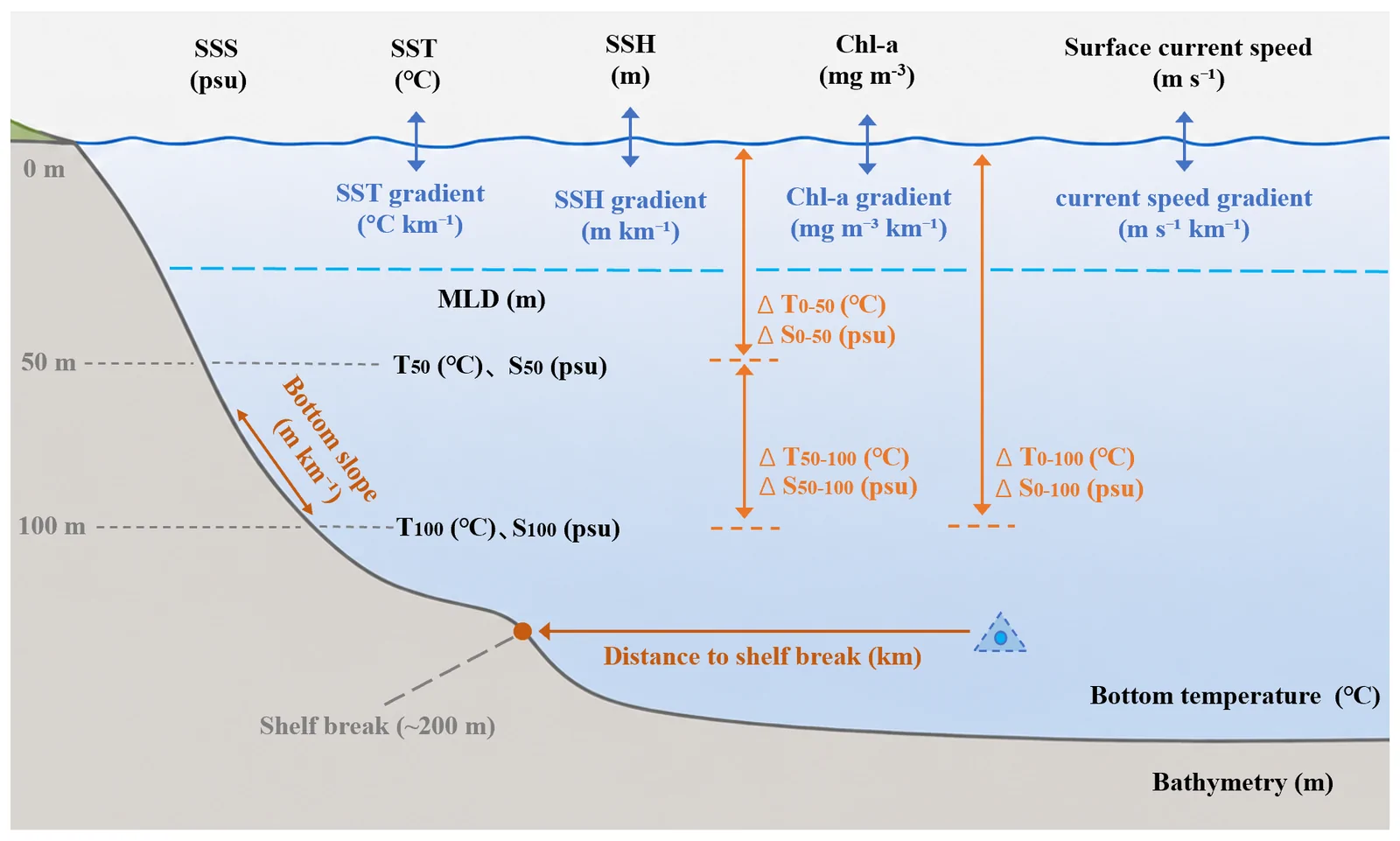
Schematic representation of the 24 candidate environmental predictors used to characterize fishing-ground conditions. The blue triangle indicates the reference point used for calculating the distance to the shelf break.

A total of 12 derived environmental variables were obtained, comprising six vertical-structure variables, two topographic-constraint variables, and four horizontal-gradient variables. Together with the 12 major environmental variables, they formed a set of 24 candidate environmental variables (Figure 2) for subsequent variable screening and model analysis.

#### 2.2.4. Variable Screening and Multicollinearity Control

**Figure 3:**
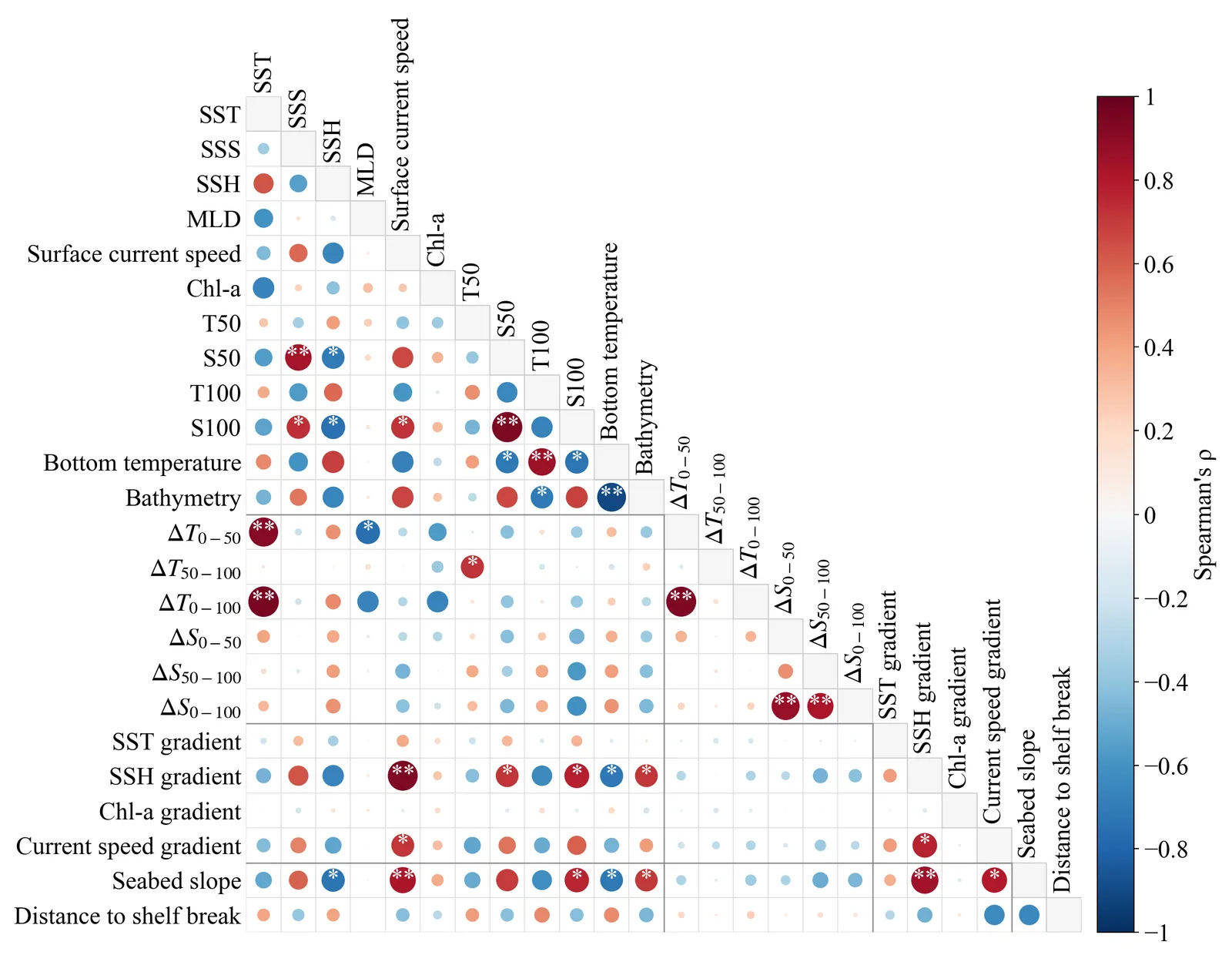
Spearman’s rank correlation matrix for the 24 candidate environmental variables. The circle size represents the absolute value of Spearman’s rank correlation coefficient (|ρ|), with larger circles indicating stronger correlations. The circle color represents the direction and strength of the correlation. (* and ** indicate 0.7 ≤ |ρ| < 0.8 and |ρ| ≥ 0.8, respectively.).

Before model fitting, the proportions of missing values and the univariate distributions of the 24 candidate environmental variables were examined, and variables with more than 15% missing values were excluded. Because fishing duration exhibited a pronounced right-skewed distribution, log(1 + fishing duration) was used as the response variable in subsequent models [30,31]. Pairwise correlations among the candidate variables were then evaluated using Spearman’s rank correlation coefficient, and variable pairs with $\left|\rho \right|\geq 0.7$ were considered highly correlated [32]. Strong correlations were observed among several temperature and salinity variables, as well as among bathymetry, bottom temperature, surface current speed, and some horizontal-gradient variables (Figure 3). In total, 32 variable pairs had $\left|\rho \right|\geq 0.7$, of which 12 had $\left|\rho \right|\geq 0.8$.

**Figure 4:**
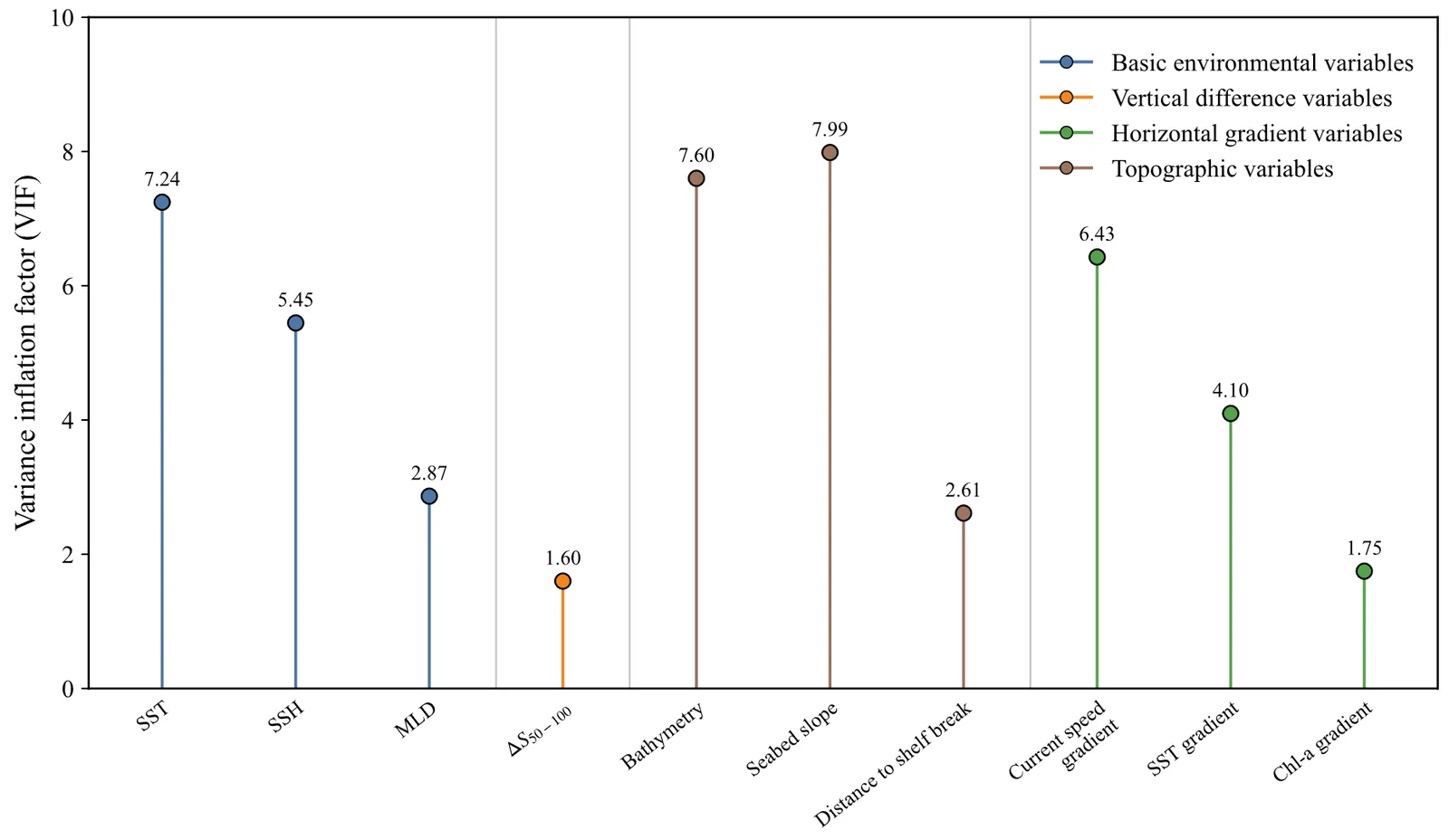
Variance inflation factors of the 10 environmental variables retained after iterative screening.

To further reduce the effects of multicollinearity, iterative screening based on the variance inflation factor (VIF) was conducted [33]. The VIF analysis was based on 28,317 complete observations, accounting for 97.14% of the total sample, and no variables with zero or near-zero variance were identified. Whenever a variable with a VIF greater than 10 was identified, variables were sequentially removed based on their correlations with other variables, proportions of missing values, and ecological relevance. VIF values were recalculated after each removal until all remaining variables had VIF values below 10. Variables with VIF values between 5 and 10 were retained but treated cautiously in subsequent interpretation [34]. After 14 screening iterations (Figure 4), 10 variables were retained: bathymetry, SST, SSH, MLD, seabed slope, distance to the shelf break, $\Delta S_{50-100}$, current speed gradient, SST gradient, and Chl-a gradient. The maximum VIF among the retained variables was 7.99.

None of the vertical temperature-difference variables were retained after VIF screening. However, given that *Illex argentinus* exhibits pronounced diel vertical movement and that temperatures at different depths and their vertical differences are associated with fishing-ground distribution [13], the potential contributions of these variables were further evaluated. Accordingly, the 10 variables retained after VIF screening (Figure 4) were designated as variable set A. $\Delta T_{0-50}$, $\Delta T_{50-100}$, and $\Delta T_{0-100}$ were then added separately to set A to form variable sets B1, B2, and B3, respectively. The performance of the four variable sets was subsequently compared using the same modeling and cross-validation procedures to determine whether a vertical temperature-difference variable should be retained.

#### 2.2.5. Model Construction and Analysis of Model-Derived Environmental Associations

A CatBoost regression model (version 1.2.10; Yandex, Moscow, Russia) implemented in Python 3.13 was first used to model the relationship between environmental variables and fishing duration [35,36]. The model parameters were set to 800 iterations, a tree depth of 6, and a learning rate of 0.05, with RMSE specified as the loss function. Fishing duration was log1p-transformed before model fitting. Leave-one-fishing-season-out (LOSO) cross-validation was used to evaluate the ability of the model to generalize across fishing seasons [37]. In each iteration, one fishing season was held out as the test set, while the remaining fishing seasons were used for model training. Missing predictor values were handled using median imputation. For each LOSO fold, predictor-specific medians were estimated using only the training data and then applied to both the training and held-out test sets, thereby avoiding information leakage. After the predictions were back-transformed to the original fishing-duration scale, model performance was evaluated using RMSE, MAE, $R^{2}$, and Spearman’s rank correlation coefficient.

Residuals from the LOSO out-of-fold predictions were further used to assess spatial and temporal autocorrelations. For the temporal autocorrelation analysis, grid-day residuals were first averaged by date to obtain a daily residual time series, and the autocorrelation function (ACF) was calculated for lags of up to 30 d. Spatial autocorrelation was evaluated using Moran’s I. Before spatial-block cross-validation, an empirical variogram of the LOSO residuals was used to estimate the approximate spatial correlation scale, defined as the first distance at which the semivariance reached 95% of the observed maximum semivariance. Candidate block sizes were then evaluated by considering both this spatial correlation scale and the resulting number and sample size of spatial blocks. Spatial-block cross-validation was then conducted to evaluate model transferability to spatially independent areas.

After the final variable set was determined based on the LOSO cross-validation results, the CatBoost model was refitted using all samples from the corresponding spatial domain. SHapley Additive exPlanations (SHAP) were then used to quantify the predictive importance of environmental variables [38], and variable importance was measured using the mean absolute SHAP value. Accumulated local effects (ALE) analysis was further used to examine the response relationships between environmental variables and predicted fishing duration [39]. For each variable, the sample distribution was divided into 20 quantile-based intervals, and the ALE curve was centered at zero. Continuous intervals with ALE values greater than zero were defined as positive-response intervals. Using the maximum ALE value (${ALE}_{max}$) as a reference, continuous intervals satisfying $ALE\left(x\right)\geq 0.90\times {ALE}_{max}$ were defined as high-response ranges [40]. When multiple continuous intervals met the high-response criterion, the interval containing the peak was retained. When $ALE_{max}\leq 0$, no high-response range was extracted. Finally, the peak location, positive-response interval, and high-response range of each major environmental predictor were obtained and used to compare model-derived environmental-response patterns between the full study area and the three subregions. These ranges represent model-derived high-response ranges identified from the ALE curves and do not represent fixed ecological thresholds. To assess the cross-season robustness of model interpretation, SHAP and ALE analyses were additionally conducted within the LOSO framework using the held-out fishing season in each fold. The stability of SHAP variable-importance rankings and the consistency of ALE response shapes across fishing seasons were then evaluated.

## 3. Results

### 3.1. Spatial Structure of Fishing-Ground Hotspots and Regionalization

**Figure 5:**
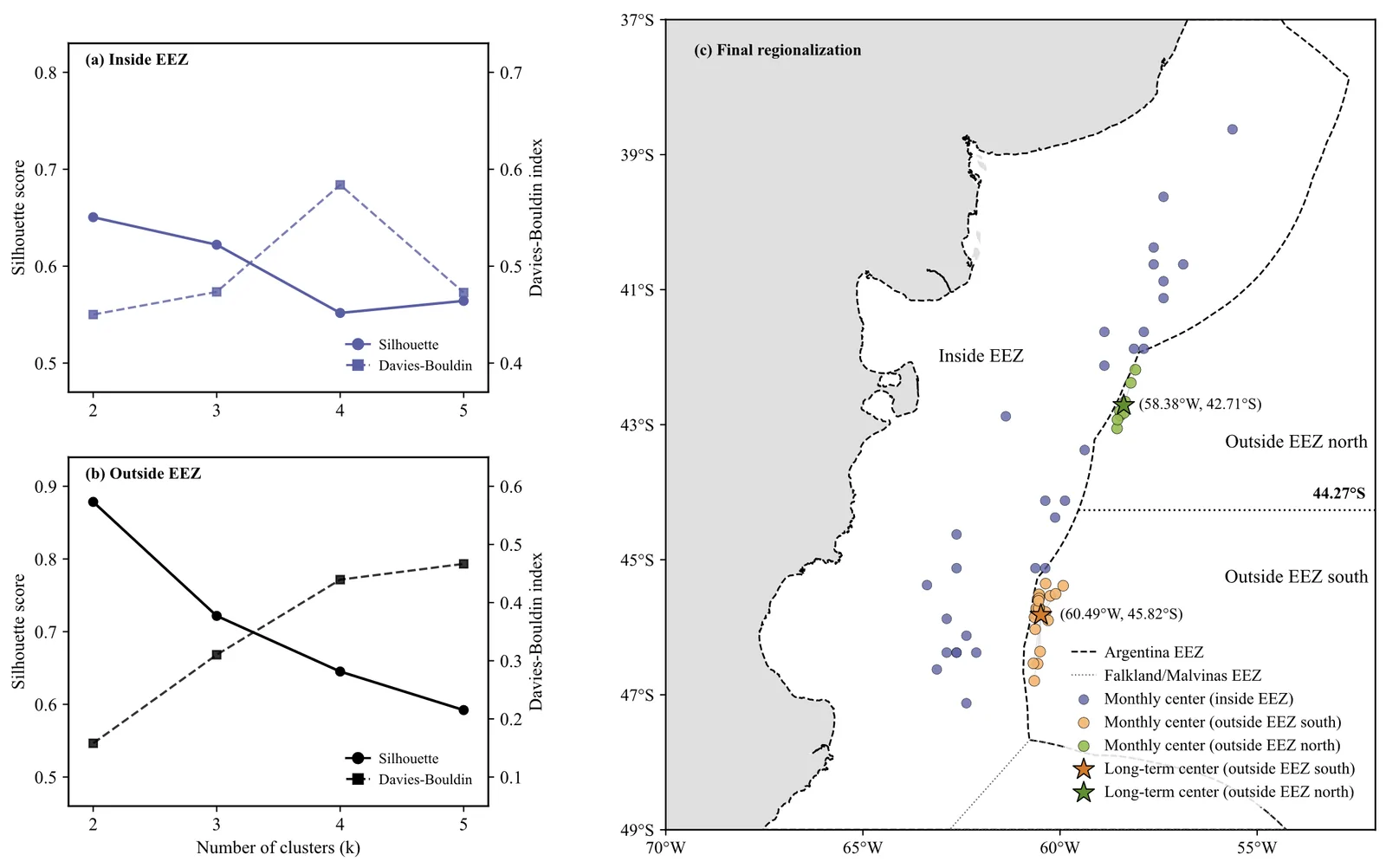
Evaluation of K-means clustering and regionalization of monthly fishing-ground centers for the Argentine shortfin squid fishery in the Southwest Atlantic. (**a**) Evaluation of K-means clustering of monthly fishing-ground centers inside the EEZ; (**b**) evaluation of K-means clustering of monthly fishing-ground centers outside the EEZ; and (**c**) final regionalization based on the spatial distribution of monthly fishing-ground centers and the long-term fishing-ground centers identified outside the EEZ.

Using the monthly fishing-ground centers derived from the 80th-percentile hotspot definition, K-means clustering of the monthly fishing-ground centers showed relatively good clustering results for both the areas inside and outside the EEZ when k=2 (Figure 5a,b). Among the tested values of k=2–5, k=2 yielded the highest silhouette scores (0.651 inside the EEZ and 0.879 outside the EEZ) and the lowest Davies–Bouldin indices (0.450 and 0.158, respectively), supporting relatively compact and well-separated two-cluster solutions. The monthly fishing-ground centers inside the EEZ formed a generally continuous spatial distribution. Although positional shifts or localized dispersion occurred in some months, they did not form two stable and clearly separated long-term fishing-ground centers (Figure 5c). The mean within-cluster dispersion radii of both potential clusters exceeded 120 km, indicating that the observed pattern mainly represented temporal shifts within a single fishing-ground range. Therefore, the fishing grounds inside the EEZ were not further divided into subregions but were retained as a single region.

The monthly fishing-ground centers outside the EEZ could be grouped into two relatively stable long-term fishing-ground centers (Figure 5c) located in the southern waters (60.49° W, 45.82° S) and the northern waters (58.38° W, 42.71° S), respectively. The distance between the two K-means centers was approximately 383.93 km, whereas the mean dispersion radii of the southern and northern clusters were only 33.92 and 27.61 km, respectively. These results indicated that the southern and northern fishing-ground centers outside the EEZ were clearly separated and that the monthly centers within each cluster were relatively concentrated. Based on the arithmetic midpoint of the latitudes of the two long-term fishing-ground centers, the boundary between the northern and southern fishing grounds outside the EEZ was defined at 44.27° S. The sensitivity analysis further showed that this regionalization was robust to the hotspot definition. Across thresholds from the 40th to the 80th percentiles, the north–south cluster assignments outside the EEZ remained unchanged, while the derived boundary latitude varied only from approximately 44.35° S to 44.27° S. The study area was ultimately divided into three subregions: waters inside the EEZ, the northern region outside the EEZ, and the southern region outside the EEZ.

### 3.2. Results for the Full-Study-Area Model

The LOSO validation results for variable sets A, B1, B2, and B3 were generally similar for the full study area (Table 1). B1 had the lowest mean RMSE (181.75 ± 29.44 h), which was 0.76% lower than that of A (183.15 ± 28.66 h), whereas its mean Spearman’s ρ (0.4483 ± 0.0880) was slightly lower than that of A (0.4485 ± 0.0903). B2 and B3 likewise showed no consistent performance advantage across the evaluation metrics. Therefore, adding $\Delta T_{0-50}$, $\Delta T_{50-100}$, or $\Delta T_{0-100}$ did not provide a consistent advantage over the baseline variable set, and the more parsimonious set A was retained as the final variable set for the full-study-area model. Under LOSO validation, the final model yielded an RMSE of 183.15 ± 28.66 h, an MAE of 74.32 ± 11.53 h, an $R^{2}$ of 0.083 ± 0.104, and a Spearman’s ρ of 0.4485 ± 0.0903, indicating limited ability to explain the absolute variation in fishing duration but some capacity to preserve relative intensity rankings.

Residual diagnostics based on the LOSO out-of-fold predictions of the final model showed weak spatial autocorrelation (Moran’s I = 0.036, *p* = 0.001), while temporal autocorrelation declined with increasing lag, with the ACF decreasing from 0.844 at 1 d to 0.128 at 30 d. The 3° spatial-block cross-validation yielded an RMSE of 206.47 h, an MAE of 81.38 h, an $R^{2}$ of −0.161, and a Spearman’s ρ of 0.072, indicating limited predictive transferability to spatially independent areas.

**Figure 6:**
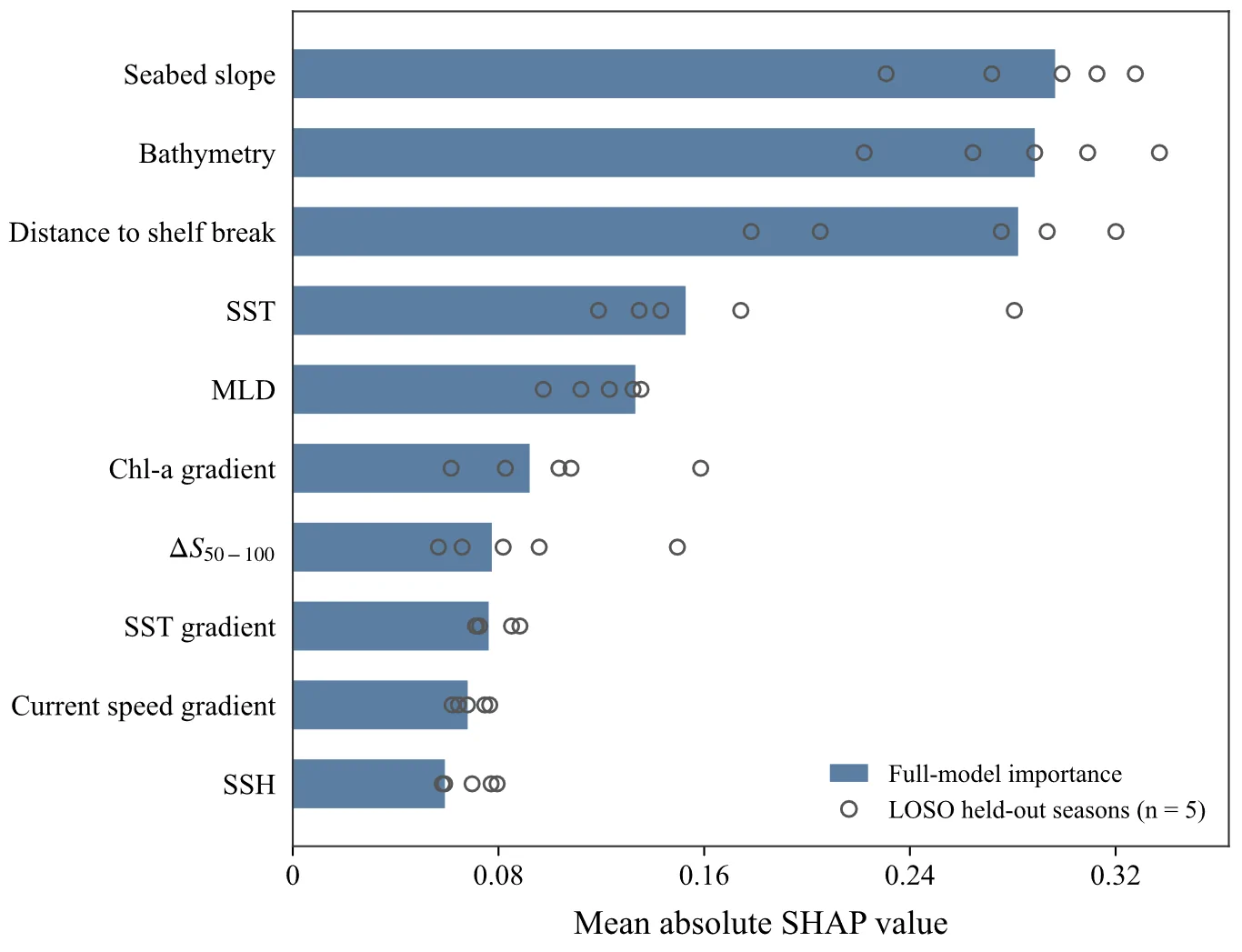
Mean absolute SHAP values and LOSO-based cross-season stability of environmental-variable predictive importance in the CatBoost model for the full study area.

SHAP analysis showed that seabed slope, bathymetry, and distance to the shelf break were the three highest-contributing environmental predictors in the full-study-area model, followed by SST and MLD, whereas the remaining variables yielded relatively lower contributions (Figure 6). LOSO out-of-fold SHAP analysis showed that variable-importance rankings were generally consistent across fishing seasons, with Spearman correlations of 0.782–0.927 relative to the formal model ranking, and topography-related variables consistently remained among the highest-ranked predictors. Overall, the full-study-area model was primarily characterized by topography-related predictors, with SST and MLD also yielding relatively high contributions.

**Figure 7:**
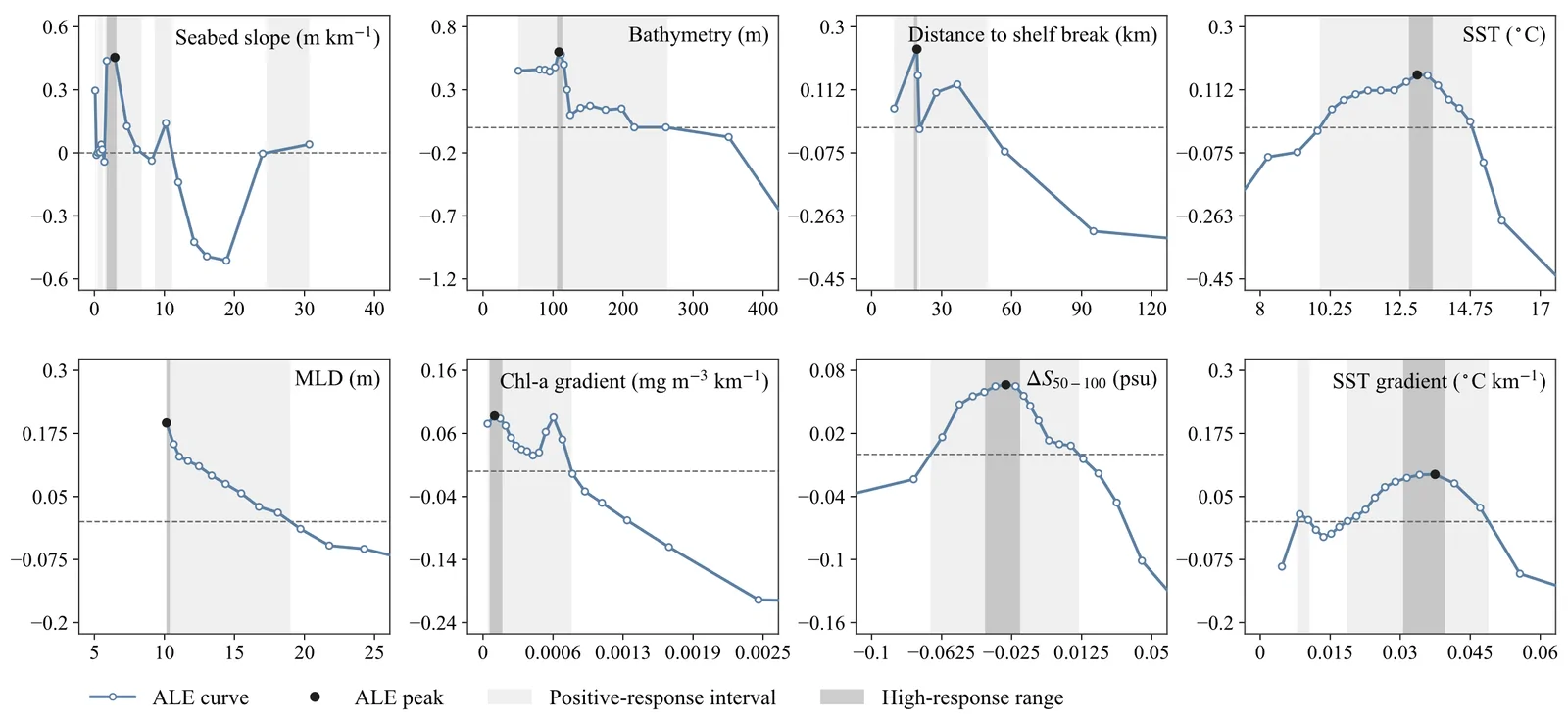
ALE response curves for the eight environmental variables with the highest mean absolute SHAP values in the CatBoost model for the full study area. The dashed horizontal line indicates ALE = 0, serving as the reference level for interpreting positive and negative ALE values. Light- and dark-gray shading indicate positive-response intervals and high-response ranges, respectively, and black points indicate ALE peaks.

Full-area ALE analysis showed nonlinear model-derived associations between the major environmental predictors and predicted fishing duration (Figure 7). The high-response ranges for seabed slope, bathymetry, and distance to the shelf break were 1.75–3.16 m km^−1^, 105.71–113.44 m, and 18.15–19.56 km, respectively, whereas those for SST and MLD were 12.78–13.54 °C and 10.15–10.39 m. The corresponding high-response ranges for the Chl-a gradient, SST gradient, and $\Delta S_{50-100}$ were 0.0001–0.0002 mg m^−3^ km^−1^, 0.0306–0.0396 °C km^−1^, and −0.0392 to −0.0204 psu, respectively. LOSO analysis indicated that the main ALE response shapes were generally consistent across fishing seasons, although some predictors showed greater interseasonal variability.

### 3.3. Results of the Subregional Models

**Figure 8:**
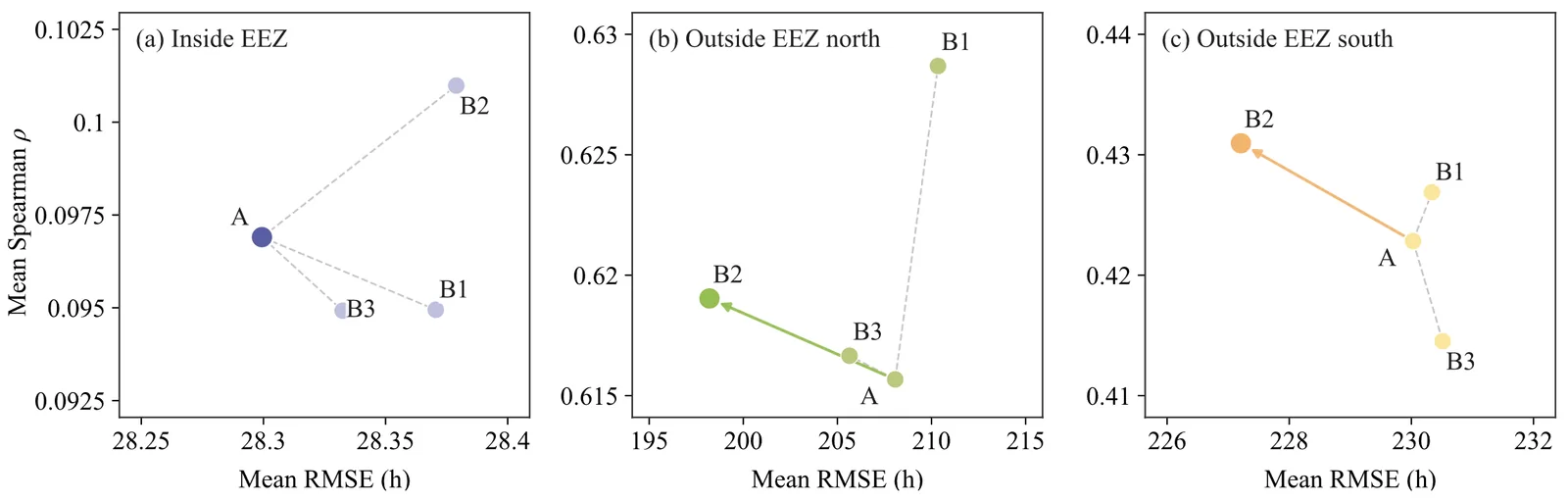
Comparison of alternative variable sets based on mean RMSE and mean Spearman’s ρ for the three subregional CatBoost models. Points closer to the upper-left indicate better overall model performance. Dashed lines connect the baseline variable set (A) with the alternative variable sets (B1, B2, and B3), and arrows indicate the direction of change relative to the baseline variable set A.

All three subregions covered five fishing seasons. The sample sizes for waters inside the EEZ, the northern region outside the EEZ, and the southern region outside the EEZ were 11,587, 3250, and 14,315, respectively. The LOSO validation results for the models corresponding to variable sets A, B1, B2, and B3 showed that the effects of including vertical temperature-difference variables varied among regions (Figure 8). Within the EEZ, variable set A yielded a relatively low mean RMSE, and the addition of any of the three vertical temperature-difference variables did not provide a consistent performance advantage across the evaluation metrics. Therefore, variable set A was selected. In the northern region outside the EEZ, the mean RMSE of B2 was 4.74% lower than that of A, while the mean Spearman’s ρ increased from 0.6157 to 0.6190. Although B1 had a higher mean Spearman’s ρ, its mean RMSE was higher than that of A; therefore, B2 was selected. In the southern region outside the EEZ, B2 likewise showed a decrease in mean RMSE and an increase in mean Spearman’s ρ, whereas B1 and B3 showed no consistent advantage. Accordingly, variable set B2, consisting of variable set A plus $\Delta T_{50-100}$, was used for both the northern and southern regions outside the EEZ.

Regional residual diagnostics showed positive spatial autocorrelation in the Inside EEZ, Outside EEZ north, and Outside EEZ south regions, with Moran’s I values of 0.316 (*p* = 0.001), 0.196 (*p* = 0.001), and 0.192 (*p* = 0.005), respectively (Table 2). Temporal autocorrelation was also present in all three regions and generally decreased with increasing time lag. Spatial-block cross-validation using 4°, 2°, and 2° blocks for the three regions yielded R^2^ values of −0.215, −0.210, and −0.228 and Spearman’s ρ values of 0.091, −0.109, and 0.095, respectively, indicating weak predictive performance in spatially independent areas (Table 3).

**Figure 9:**
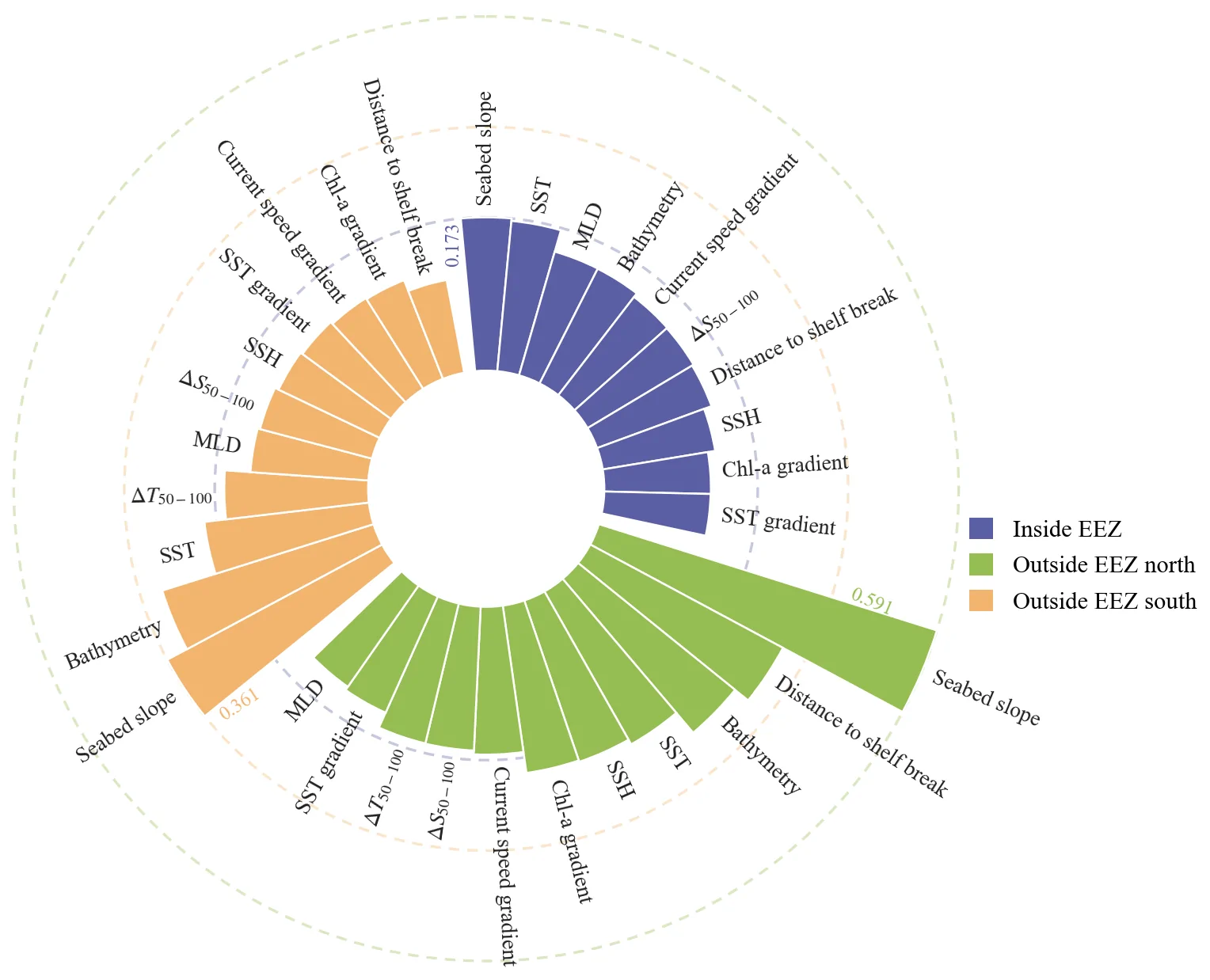
Environmental-variable predictive importance in the three regional CatBoost models based on mean absolute SHAP values. Dashed circles indicate the maximum mean absolute SHAP value within each region and provide reference scales for comparing the relative importance of environmental variables within each regional model.

SHAP analysis showed differences in variable-importance rankings and major predictors among the regional models (Figure 9). Within the EEZ, the major contributors included seabed slope, SST, MLD, and bathymetry. In the northern region outside the EEZ, the major predictors included topography-related variables, such as seabed slope, distance to the shelf break, and bathymetry. In the southern region outside the EEZ, seabed slope, bathymetry, and SST showed relatively high predictive importance, while the vertical-structure variable $\Delta T_{50-100}$ also ranked among the major predictors. LOSO results further showed that the stability of variable-importance rankings differed among predictors (Table 4). Seabed slope showed no rank variation across the five LOSO folds in the northern region (SD = 0), while bathymetry, distance to the shelf break, and $\Delta T_{50-100}$ showed relatively small rank variation in the southern region. By contrast, greater cross-season variation occurred for several predictors within the EEZ.

**Figure 10:**
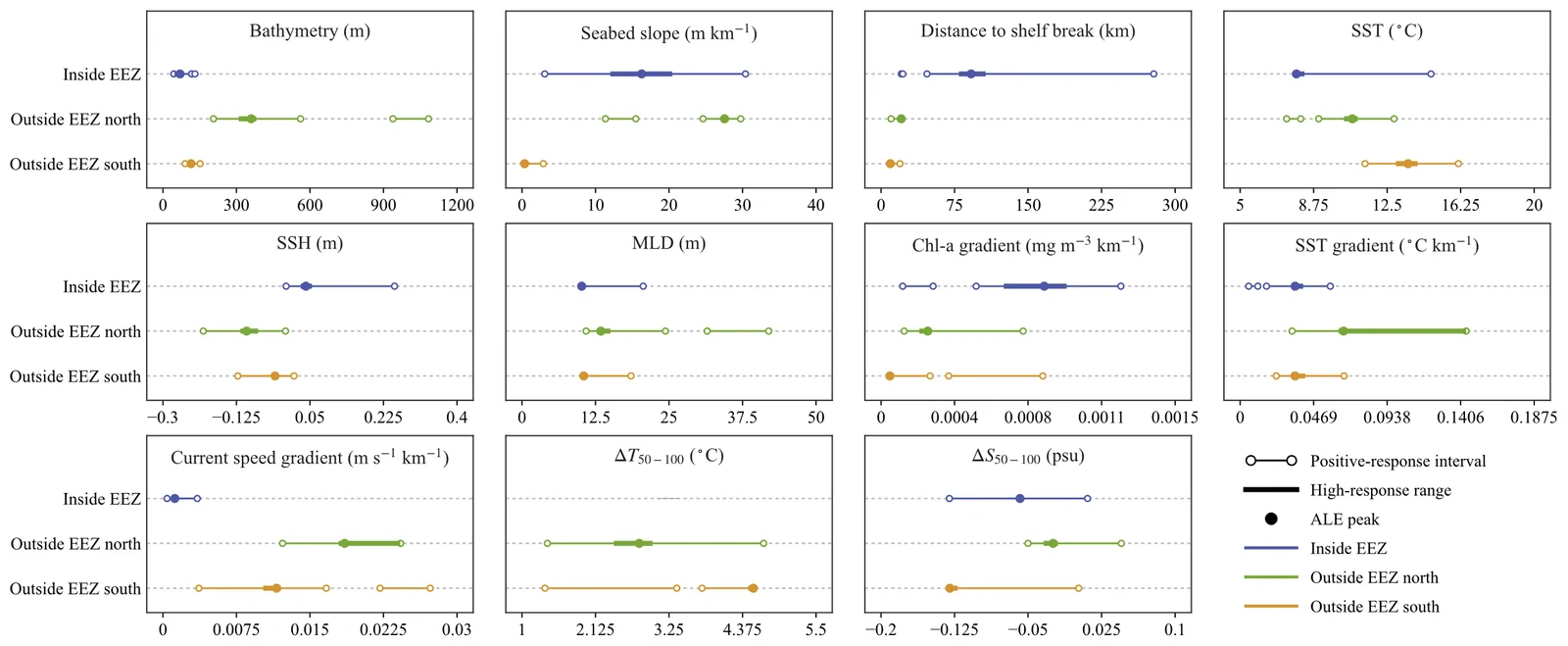
Regional differences in ALE-derived response ranges of environmental variables.

The response characteristics and high-response ranges of the major environmental predictors differed among the three regions (Figure 10). Within the EEZ, higher predicted fishing duration was mainly associated with lower SST and greater distance to the shelf break. In the northern region outside the EEZ, higher predicted fishing duration was mainly associated with deeper waters and steeper seabed slopes, whereas the southern region outside the EEZ was characterized by shallower water, gentler seabed slopes, and clearly defined response ranges for vertical temperature and salinity structure.

Within the EEZ, the high-response ranges were 12.01–20.44 m km^−1^ for seabed slope, 7.87–8.28 °C for SST, 10.15–10.44 m for MLD, and 47.04–72.84 m for bathymetry. Distance to the shelf break showed two positive-response intervals, with a high-response range of 79.41–106.72 km. Overall, higher predicted fishing duration within the EEZ was mainly associated with steeper seabed slopes, lower SST, shallower water, and greater distance to the shelf break.

In the northern region outside the EEZ, the high-response range for seabed slope was 27.41–27.77 m km^−1^, while those for distance to the shelf break and bathymetry were 20.52–20.58 km and 309.11–381.13 m, respectively. The high-response ranges for SST and SSH were 10.30–11.00 °C and −0.116 to −0.074 m, respectively. The Chl-a gradient had a high-response range of 0.0002–0.0003 mg m^−3^ km^−1^, while that of $\Delta T_{50-100}$ was 2.41–3.00 °C. Compared to waters inside the EEZ, higher predicted fishing duration in the northern region outside the EEZ was mainly associated with locations closer to the shelf break, greater water depth, and steeper seabed slopes.

In the southern region outside the EEZ, the high-response ranges for seabed slope and bathymetry were 0.36–0.42 m km^−1^ and 104.97–116.93 m, respectively, while that for SST was 12.95–14.05 °C. The high-response ranges for $\Delta T_{50-100}$ and $\Delta S_{50-100}$ were 4.46–4.54 °C and −0.130 to −0.122 psu, respectively, and that for MLD was 10.50–10.52 m. The high-response range for distance to the shelf break was 9.46–10.87 km. Compared to the northern region outside the EEZ, the high-response ranges in the southern region outside the EEZ corresponded to shallower water and gentler seabed slopes. In addition, both $\Delta T_{50-100}$ and $\Delta S_{50-100}$ showed clearly defined high-response ranges.

LOSO analysis further showed that the ALE response shapes of most environmental predictors showed some degree of consistency across fishing seasons (Table 5). MLD showed consistently high agreement across all three regions (ρ = 0.90–0.98), while bathymetry also exhibited relatively stable response shapes (ρ = 0.86–0.99). By contrast, lower cross-season consistency was observed for some predictors, such as SST in the southern region outside the EEZ (ρ = 0.48) and ΔS_50_–_100_ in the northern region outside the EEZ (ρ = 0.50).

## 4. Discussion

### 4.1. Fishing-Ground Regionalization

The fishing grounds of *Illex argentinus* show pronounced seasonal variation, which is closely related to the migratory processes of different stocks. During the early and middle stages of the fishing season, the Summer-Spawning Stock migrates from the central shelf toward the outer shelf, and the high-seas shelf area near 44° S–47°30′ S becomes the main fishing area. From April to June, part of the Bonaerensis–North Patagonian Stock (BNS) migrates northeastward and forms a northern fishing ground near 41° S–42° S [41,42]. The distribution of *Illex argentinus* resources within the EEZ exhibits a north–south spatial structure, and the area near 44° S is generally regarded as an important latitudinal boundary separating the South Patagonian Stock (SPS) from the BNS [20].

At k=2, the monthly fishing-ground centers within the EEZ showed a relatively clear clustering pattern. The two potential centers were located at 61.90° W, 45.42° S and 57.76° W, 41.11° S, respectively, on opposite sides of 44°S. This distribution was generally consistent with the previously reported latitudinal spatial pattern (Figure 5b). However, a single main fishing core remained dominant in most months with valid data within the EEZ, and the mean dispersion radius of each potential cluster exceeded 120 km, indicating considerable variation in the locations of the monthly fishing-ground centers within the clusters. Therefore, these two centers were more likely to reflect north–south differences in the principal fishing locations during different stages of the fishing season than two spatially stable independent fishing grounds with clearly defined boundaries. Further dividing the EEZ into subregions according to these two potential centers and fitting separate models could result in insufficient data to support individual subregional models across different months and fishing seasons. Therefore, waters within the EEZ were retained as a single region for the subsequent environmental-response analysis.

By contrast, the monthly fishing-ground centers outside the EEZ formed a clearer north–south dual-center structure (Figure 5c). This pattern was generally consistent with the previously reported distribution of high-seas trawl fishing grounds for *Illex argentinus*, with the southern fishing ground located mainly near 45° S and the northern fishing ground generally appearing near 42° S after April [9]. Moreover, the two fishing-ground centers recurred across multiple fishing seasons, indicating that the southern and northern waters outside the EEZ contained relatively stable long-term areas of fishing concentration. Therefore, based on the spatial distribution of the monthly fishing-ground centers, waters outside the EEZ were divided into northern and southern regions at 44.27° S for subsequent comparisons of regional environmental responses. However, the resulting differences among regional models should not be attributed solely to environmental heterogeneity. Fishing operations within and outside the EEZ also differ in access authorization, operational management, and fleet composition, and these fishery-related differences may influence fishing duration and contribute to the observed regional patterns.

### 4.2. Identification of Major Environmental Predictors

Seabed slope, bathymetry, and SST yielded relatively high predictive contributions to the model for the full study area and in all three regional models. Seabed slope ranked first in every model, while bathymetry and SST consistently ranked among the four highest-ranked predictors (Figure 6 and Figure 9). Because the three regional models were based on unequal sample sizes and different fishing-duration distributions, regional SHAP results were interpreted primarily according to within-model variable rankings rather than by directly comparing absolute mean SHAP magnitudes across regions. Accordingly, SHAP importance in this study represents predictive importance within the fitted models and should not be interpreted as the biological importance of individual environmental variables. *Illex argentinus* mainly aggregates over the central and outer Patagonian Shelf, and its abundance varies seasonally among different depth ranges [6]. Bathymetry indicates not only water depth but also the position of fishing grounds on the continental shelf. Unlike bathymetry, seabed slope characterizes the rate of local change in water depth and therefore reflects topographic transition zones. Multiple thermal fronts commonly occur near the Patagonian shelf break, and off-shelf waters associated with the Falkland Current may intrude onto the shelf, altering the local distributions of temperature, salinity, and Chl-a [17]. These processes may make areas with stronger bathymetric gradients more likely to develop water-mass boundaries and frontal environments, and squid-jigging operations targeting *Illex argentinus* are also frequently concentrated near these frontal zones [15]. Therefore, seabed slope does not necessarily directly represent the physiological requirements of *Illex argentinus* but may instead serve as a proxy for local topographic and current conditions associated with fishing-ground use. Although bathymetry and seabed slope contain some overlapping information, the performance of the full-study-area model declined after seabed slope was removed, suggesting that seabed slope provided predictive information on local topographic variation that was not fully captured by bathymetry and distance to the shelf break. SST also maintained a relatively high predictive contribution to the model for the full study area and in all regional models, indicating that surface water temperature is a relatively high-ranked environmental predictor associated with spatial variation in fishing duration for *Illex argentinus*. The species has a short life cycle and pronounced population fluctuations, and temperature variation can affect its body size, maturation process, and abundance [2]. In addition, the convergence of the Brazil Current and the Falkland Current creates pronounced temperature differences and frontal structures. Research on high-seas trawl fishing grounds has also shown that SST is a key environmental factor during most 10-day periods [9].

During variable screening (Figure 3), Chl-a itself was not retained in the final model, whereas the Chl-a gradient was retained. The retention of the Chl-a gradient indicates that spatial variation in Chl-a provides predictive information to the final model and may be associated with environmental transition zones. Chl-a shows pronounced spatial variation over the Patagonian Shelf and in the shelf-break region, and clear water-mass boundaries and productivity differences frequently occur near frontal zones [16]. Adult *Illex argentinus* mainly feed on crustaceans, amphipods, and krill [7]. One possible explanation is that the Chl-a gradient is associated with spatial variations in primary productivity and prey conditions that coincide with fishing-ground use. However, primary productivity and prey abundance were not directly measured in this study; therefore, this interpretation should be regarded as a possible ecological explanation rather than a demonstrated mechanism. Similarly, salinity at individual depths was not retained, whereas $\Delta S_{50-100}$ was included in the final variable sets for the full study area and all three subregions. The retention of $\Delta S_{50-100}$ suggests that the vertical salinity structure provides predictive information that is not represented by salinity at a single depth. *Illex argentinus* migrates between the continental shelf and slope, and its migration pathways are associated with the density structure of shelf and offshore waters [5]. Because seawater density is jointly influenced by temperature and salinity, the retention of $\Delta S_{50-100}$ reflects differences in water masses, stratification, or exchange processes between shelf and offshore waters within the 50–100 m layer. However, these water-mass and stratification processes were not directly quantified in the present analysis; therefore, their roles remain possible explanations for the observed model-derived association rather than demonstrated causal pathways. The SST gradient and current speed gradient were also retained, suggesting that the spatial variation in environmental conditions provides supplementary predictive information in the models. However, the SST gradient had a relatively low mean SHAP ranking in all models, suggesting that it primarily provides supplementary information on local fronts or water-mass transitions rather than acting as a dominant predictor associated with spatial variation in high fishing duration.

### 4.3. Spatial Heterogeneity of Model-Derived Environmental Associations

The high-response ranges of the major environmental predictors shared among models were not consistent across regions, indicating that higher fishing duration did not occur under identical topographic and temperature conditions. Because regional sample sizes differed, comparisons among regions were based primarily on within-model predictor rankings and ALE response patterns rather than absolute SHAP magnitudes. Taking bathymetry and seabed slope as examples, waters inside the EEZ, the southern region outside the EEZ, and the northern region outside the EEZ corresponded to fishing settings on the shallow shelf, the outer shelf, and near the deeper continental slope, respectively. Previous research on the northern Patagonian Shelf showed that areas of high *Illex argentinus* abundance occurred within different depth ranges among months, mainly at depths of 100–400 m from March to April and 80–200 m from May to June [6]. Therefore, the bathymetric and seabed-slope ranges identified for different regions mainly reflect differences in fishing locations and seasonal shifts in fishing grounds and should not be interpreted as fixed ecological thresholds. The high-response range for bathymetry across the full study area was relatively similar to that in the southern region outside the EEZ, possibly because of the larger sample size and greater concentration of fishing activity in the southern high seas. However, the response estimated by the full-study-area model represented the combined patterns of multiple regions.

The high-response ranges for SST across the full study area and the southern and northern regions outside the EEZ were concentrated between 10.30 and 14.05 °C, whereas the corresponding range inside the EEZ was 7.87–8.28 °C. Overall, these ranges were consistent with previously reported SST conditions for *Illex argentinus* fishing grounds in the Southwest Atlantic. Based on fishery data from different years and fishing methods, SST in the principal *Illex argentinus* fishing grounds in this region has generally ranged from approximately 7 to 15 °C. Reported suitable SST ranges for squid-jigging fishing grounds include 7–15 °C and 9–14 °C, while that for high-seas trawl fishing grounds over the entire fishing season is 8.2–14.6 °C [9,43,44]. Although the high-response SST ranges extracted for all regions fell within the previously reported temperature conditions of the fishing grounds, the range inside the EEZ was clearly at the lower end. Squid-jigging fishery data collected inside the Argentine EEZ from 2008 to 2011 showed that the suitable SST range in the central fishing grounds gradually decreased from 11–14.5 °C in February to 7–9 °C in June [45]. The lower high-response SST range inside the EEZ was consistent with the low-temperature fishing window during the later part of the fishing season in this region. Given the smaller sample size inside the EEZ and the restriction of fishing locations by management boundaries, this range may primarily reflect the surface temperature conditions associated with local records of higher fishing duration.

Regarding the vertical temperature structure, neither $\Delta T_{0-50}$ nor $\Delta T_{0-100}$ were retained in the final variable sets for the full study area or any of the subregions, whereas $\Delta T_{50-100}$ was retained for both the northern and southern regions outside the EEZ. This result suggests that the thermal structure within the 50–100 m layer provides additional predictive information mainly for the high-seas fishing areas. *Illex argentinus* is generally distributed within the 50–200 m water layer, and environmental conditions below 50 m are associated with its spatial distribution [13]. Its migration between the continental shelf and slope is also closely associated with the water-column density structure and water-mass exchange processes [5]. The high-seas fishing grounds off Argentina are located near the outer shelf and shelf break and are frequently influenced by the Falkland Current, shelf waters, and off-shelf waters. In addition, the Patagonian shelf-break front has a pronounced vertical structure, with its inner boundary located approximately along the 90–100 m isobaths. Off-shelf waters may also intrude onto the northern Patagonian Shelf and alter the local temperature and salinity structure [17]. Therefore, the retention of $\Delta T_{50-100}$ likely reflects the relationship between high fishing duration and the hydrographic structure of the shelf-break region. $\Delta T_{50-100}$ ranked fourth within the southern regional model but ranked considerably lower within the northern regional model, possibly due to differences in bathymetric conditions between the two regions (Figure 9). The high-response bathymetric range in the southern region outside the EEZ was 105–117 m, which was relatively close to the 50–200 m layer commonly used by *Illex argentinus* and to the 90–100 m isobaths near the inner boundary of the Patagonian shelf-break front [13,17]. By contrast, the high-response bathymetric range in the northern region outside the EEZ was 309–381 m; thus, $\Delta T_{50-100}$ is less representative of the deeper fishing setting in this region than in the southern region outside the EEZ. This variable was not retained for the full study area, possibly because it encompassed multiple fishing settings, including the shallow shelf, shelf break, and deeper continental slope. Consequently, the regional predictive pattern of $\Delta T_{50-100}$ observed in the high-seas regions was weakened when the data were combined in a single model. Inside the EEZ, the high-response bathymetric range was relatively shallow, and fishing locations were constrained by management boundaries. Therefore, $\Delta T_{50-100}$ is less representative of the principal fishing environment than SST, MLD, bathymetry, and seabed slope.

MLD ranked among the five highest-ranked predictors in the full study area, inside the EEZ, and in the southern region outside the EEZ, but it had a lower within-model ranking in the northern region outside the EEZ (Figure 6 and Figure 9). MLD represents the depth of vertical mixing in the upper ocean and is associated with surface heat exchange, nutrient exchange, and the vertical structure of the water column [46]. Previous potential-distribution models for *Illex argentinus* also identified MLD as an important environmental variable, suggesting that the mixed-layer structure is associated with the distribution of its fishing grounds [47]. The high-response ranges for MLD across the full study area, inside the EEZ, and in the southern region outside the EEZ were 10.15–10.39 m, 10.15–10.44 m, and 10.50–10.52 m, respectively. These values correspond to relatively shallow mixed-layer conditions. Although they were slightly lower than the range of 12.9–26.1 m reported in other models of high-seas resource abundance, they remained within an overall shallow mixed-layer setting. These differences may be related to differences in fishing methods, response variables, and regionalization scales [48]. MLD did not rank among the five highest-ranked predictors in the northern region outside the EEZ, possibly because fishing operations in this region occurred in deeper waters. Because MLD mainly describes the thickness of the near-surface mixed layer, it does not fully represent environmental differences under deeper continental-slope conditions, resulting in a lower relative predictive ranking than in the other regions.

The current speed gradient showed relatively high within-model ranking only inside the EEZ, whereas surface current speed itself was not retained during variable screening. Surface current speed represents the magnitude of seawater flow, whereas the current speed gradient more directly reflects changes in current speed between adjacent waters and is therefore more suitable for characterizing hydrodynamic variation near flow-field transition zones, water-mass boundaries, and fronts over the shallow shelf. Various fronts occur over the Argentine Shelf, and their formation is associated with tides, river discharge, current convergence, topography, and water-column mixing [15]. Fishing activity is also frequently concentrated near these fronts [16]. Therefore, the relatively high predictive ranking of the current speed gradient inside the EEZ indicates that higher fishing duration in this region was more strongly associated with areas of pronounced local variation in the flow field than with current speed alone. These areas coincide with water-mass boundaries, prey distribution, and fishing locations, providing a possible environmental context for the spatial distribution of fishing duration for *Illex argentinus*.

In addition, SSH displayed relatively low within-model rankings across the full study area, inside the EEZ, and in the southern region outside the EEZ, but it was relatively important in the northern region outside the EEZ. This suggests that, in addition to topography and SST, higher fishing duration in the northern region outside the EEZ is also associated with hydrodynamic conditions reflected by sea surface height. The high-seas fishing grounds of *Illex argentinus* are located near the convergence zone of the Falkland Current and the Brazil Current, where changes in sea surface height are commonly associated with current convergence and divergence, eddy activity, and upwelling or downwelling [49,50]. In some fishing-ground studies, lower sea surface height or negative sea surface height anomalies have been used to indicate upwelling, cold eddies, or nutrient-supply processes, which are associated with conditions favorable for phytoplankton and prey aggregation [51]. By contrast, the full-study-area model combined samples from multiple regions, while higher fishing duration inside the EEZ and in the southern region outside the EEZ was more strongly associated in the models with variables such as bathymetry, SST, and MLD. Consequently, SSH exhibited relatively lower predictive rankings in those models.

## 5. Conclusions

Daily fishing duration extracted from VMS position data can characterize fishing-ground use intensity by the *Illex argentinus* jigging fleet at a relatively high spatiotemporal resolution and describe its relationship with the marine environment. Although the models showed limited ability to predict absolute variation in fishing duration, they retained some capability to distinguish relative fishing-activity intensity and revealed regional differences in model-derived environmental associations. Seabed slope, bathymetry, and SST yielded relatively high predictive contributions across the full study area and all subregions. Their high-response ranges were generally consistent with the environmental conditions reported in previous fishing-ground studies, indicating that VMS-derived fishing duration can provide useful information about fishing-ground use patterns. It should be emphasized that these ranges represent model-response intervals associated with higher fishing duration and should not be interpreted as fixed ecological or physiological thresholds for *Illex argentinus*. The inclusion of derived environmental variables provided additional information on local topography, vertical water-column structure, and environmental transitions. Distance to the shelf break, $\Delta T_{50-100}$, $\Delta S_{50-100}$, and horizontal-gradient variables yielded different predictive contributions among regions. Using 44.27° S as the boundary of the fishing-ground use pattern outside the EEZ, waters outside the EEZ were divided into northern and southern fishing grounds, which, together with waters inside the EEZ, formed three subregions. The subregional models showed that waters inside the EEZ were characterized by a composite shallow-shelf association pattern, with relatively prominent contributions from SST, MLD, and the current speed gradient. The northern region outside the EEZ showed a strong shelf-break-proximity pattern and an association with hydrodynamic conditions reflected by SSH. The southern region outside the EEZ mainly reflected the combined predictive contributions of topography, SST, and the vertical temperature structure between 50 and 100 m. By comparison, the full-study-area model represented an integrated pattern across multiple fishing settings. Although it identified common major environmental predictors, including seabed slope, bathymetry, and SST, it also weakened or obscured region-specific environmental associations. Therefore, the model-derived environmental associations with higher fishing duration differed among regions, despite the limited absolute predictive performance of the models. These findings suggest that fishing-ground monitoring and management should not rely solely on a single model fitted to the full study area. Instead, region-specific monitoring indicators should be developed according to the dominant predictive patterns in different regions to support the identification of high-use fishing grounds and spatially refined management.

## Figures and Tables

**Table 1:** LOSO validation performance of CatBoost models using alternative variable sets for the full study area.

Variable Set	Variable-Set Modification	RMSE (h)	ΔRMSE vs. A (%)	MAE (h)	$R^{2}$	Spearman’s ρ
A	Baseline variable set	183.15 ± 28.66	—	74.32 ± 11.53	0.083 ± 0.104	0.4485 ± 0.0903
B1	A + $\Delta T_{0-50}$	181.75 ± 29.44	−0.76	74.02 ± 11.62	0.098 ± 0.106	0.4483 ± 0.0880
B2	A + $\Delta T_{50-100}$	182.39 ± 30.35	−0.41	74.28 ± 12.13	0.093 ± 0.103	0.4407 ± 0.0858
B3	A + $\Delta T_{0-100}$	184.12 ± 29.58	+0.53	74.83 ± 11.85	0.073 ± 0.115	0.4428 ± 0.0848

Notes: Values represent the mean ± SD across the five leave-one-fishing-season-out folds. ΔRMSE was calculated relative to variable set A; the dash (—) indicates that ΔRMSE was not calculated for the baseline variable set A because it was compared with itself. negative values indicate a lower RMSE than the baseline variable set.

**Table 2:** Predictive performance of the final regional CatBoost models under leave-one-season-out cross-validation.

Region	RMSE (h)	MAE (h)	R^2^	Spearman’s ρ
Inside EEZ	28.32 ± 5.80	16.61 ± 3.42	−0.211 ± 0.135	0.092 ± 0.053
Outside EEZ north	199.05 ± 81.77	91.49 ± 28.60	−0.212 ± 0.928	0.623 ± 0.083
Outside EEZ south	226.39 ± 22.32	113.31 ± 8.79	0.051 ± 0.095	0.434 ± 0.087

**Table 3:** Predictive performance of the final regional CatBoost models under spatial-block cross-validation.

Region	Block Size	RMSE (h)	MAE (h)	R^2^	Spearman’s ρ
Inside EEZ	4°	28.92	16.14	−0.215	0.091
Outside EEZ north	2°	268.90	114.77	−0.210	−0.109
Outside EEZ south	2°	259.11	123.58	−0.228	0.095

**Table 4:** Cross-season variability in SHAP variable-importance rankings across the three subregions.

Environmental Variable	Inside EEZ	Outside EEZ North	Outside EEZ South
Bathymetry	2.387	1.140	0.447
Seabed slope	2.074	0.000	0.894
Distance to shelf break	1.140	0.837	0.447
SST	1.140	2.793	1.517
SSH	2.966	2.588	2.049
MLD	2.608	2.387	1.225
Chl-a gradient	2.345	2.074	2.345
SST gradient	1.517	1.414	0.837
Current speed gradient	1.673	1.140	2.000
$\Delta T_{50-100}$	—	2.550	0.548
$\Delta S_{50-100}$	3.362	2.168	2.074

Notes: Values represent the standard deviation of SHAP importance ranks across the five LOSO held-out fishing seasons. Lower values indicate more stable cross-season rankings. “—” indicates that the predictor was not included in the corresponding regional model.

**Table 5:** Cross-season consistency of ALE response shapes for environmental predictors across the three subregions.

Environmental Variable	Inside EEZ	Outside EEZ North	Outside EEZ South
Bathymetry	0.86	0.97	0.99
Seabed slope	0.91	0.77	0.88
Distance to shelf break	0.74	0.95	0.56
SST	0.75	0.65	0.48
SSH	0.85	0.89	0.54
MLD	0.98	0.90	0.93
Chl-a gradient	0.64	0.87	0.55
SST gradient	0.62	0.83	0.91
Current speed gradient	0.98	0.86	0.57
$\Delta T_{50-100}$	—	0.83	0.56
$\Delta S_{50-100}$	0.71	0.50	0.66

Notes: Values represent the mean Spearman’s rank correlation coefficients between the ALE response curve from the formal full-data regional model and the corresponding curves from the five LOSO held-out fishing seasons. Higher values indicate greater cross-season consistency in response shape. “—” indicates that the predictor was not included in the corresponding regional model.

## Data Availability

The marine environmental data used in this study can be downloaded from the following websites: “https://data.marine.copernicus.eu/products (accessed on 5 February 2026)” and “https://cds.climate.copernicus.eu/datasets (accessed on 13 November 2025)”. The Vessel Monitoring System data are not publicly available because of confidentiality restrictions imposed by the data provider.

## References

[1] Büring T., Baylis A., Brickle P. (2025). Analysing and Forecasting Spatio-Temporal Abundance of *Illex argentinus* in the Falkland Islands Zone Based on Oceanographic Features. Estuar. Coast. Shelf Sci..

[2] Chemshirova I., Hoving H.-J., Arkhipkin A. (2021). Temperature Effects on Size, Maturity, and Abundance of the Squid *Illex argentinus* (Cephalopoda, Ommastrephidae) on the Patagonian Shelf. Estuar. Coast. Shelf Sci..

[3] Waluda C.M., Griffiths H.J., Rodhouse P.G. (2008). Remotely Sensed Spatial Dynamics of the *Illex argentinus* Fishery, Southwest Atlantic. Fish. Res..

[4] Rodhouse P.G.K. (2001). Managing and Forecasting Squid Fisheries in Variable Environments. Fish. Res..

[5] Arkhipkin A.I., Gras M., Blake A. (2015). Water Density Pathways for Shelf/Slope Migrations of Squid *Illex argentinus* in the Southwest Atlantic. Fish. Res..

[6] Bazzino G., Quiñones R.A., Norbis W. (2005). Environmental Associations of Shortfin Squid *Illex argentinus* (Cephalopoda: Ommastrephidae) in the Northern Patagonian Shelf. Fish. Res..

[7] Mouat B., Collins M.A., Pompert J. (2001). Patterns in the Diet of *Illex argentinus* (Cephalopoda: Ommastrephidae) from the Falkland Islands Jigging Fishery. Fish. Res..

[8] Xiang D., Sun Y., Zhu H., Wang J., Huang S., Zhang S., Zhang F., Zhang H. (2025). Comparative Analysis of Prediction Models for Trawling Grounds of the Argentine Shortfin Squid *Illex Argentinus* in the Southwest Atlantic High Seas Based on Vessel Position and Fishing Log Data. Biology.

[9] Xiang D., Li Y., Jiang K., Han H., Wang Y., Yang S., Zhang H., Sun Y. (2024). Environmental Influences on *Illex argentinus* Trawling Grounds in the Southwest Atlantic High Seas. Fishes.

[10] Liao H., Jin P., Feng Z., Ji Z., Cao J., Yu W. (2025). Impacts of Marine Heatwaves on the Abundance and Distribution of *Illex argentinus* on the Patagonian Shelf. Rev. Fish Biol. Fish..

[11] Gerritsen H., Lordan C. (2011). Integrating Vessel Monitoring Systems (VMS) Data with Daily Catch Data from Logbooks to Explore the Spatial Distribution of Catch and Effort at High Resolution. ICES J. Mar. Sci..

[12] Li G., Lu Z., Cao Y., Zou L., Chen X. (2023). CPUE Estimation and Standardization Based on VMS: A Case Study for Squid-Jigging Fishery in the Equatorial of Eastern Pacific Ocean. Fishes.

[13] Liu H., Yu W., Chen X. (2022). Melting Antarctic Sea Ice Is Yielding Adverse Effects on a Short-Lived Squid Species in the Antarctic Adjacent Waters. Front. Mar. Sci..

[14] Shang C., Han H., Chen J., Tang F., Fan W., Zhang H., Cui X. (2023). Relationship between Resource Distribution and Vertical Structure of Water Temperature of Purpleback Flying Squid (*Sthenoteuthis Oualaniensis*) in the Northwest Indian Ocean Based on GAM and GBT Models. J. Mar. Sci. Eng..

[15] Acha E.M., Mianzan H.W., Guerrero R.A., Favero M., Bava J. (2004). Marine Fronts at the Continental Shelves of Austral South America: Physical and Ecological Processes. J. Mar. Syst..

[16] Alemany D., Acha E.M., Iribarne O.O. (2014). Marine Fronts Are Important Fishing Areas for Demersal Species at the Argentine Sea (Southwest Atlantic Ocean). J. Sea Res..

[17] Franco B.C., Piola A.R., Rivas A.L., Baldoni A., Pisoni J.P. (2008). Multiple Thermal Fronts near the Patagonian Shelf Break. Geophys. Res. Lett..

[18] Rodhouse P.G., Barton J., Hatfield E.M.C., Symon C. (1995). *Illex Argentinus*: Life Cycle, Population Structure, and Fishery. ICES Mar. Sci. Symp..

[19] Harte M., Borberg J., Sylvia G. (2018). Argentine Shortfin Squid (Illex Argentinus) Value Chain Analysis with an Emphasis on the Falkland Islands.

[20] Haimovici M., Brunetti N.E., Rodhouse P.G., Csirke J., Leta R.H., Rodhouse P.G., Dawe E.G., O’Dor R.K. (1998). *Illex* *Argentinus*. Squid Recruitment Dynamics: The Genus Illex as a Model, the Commercial Illex Species and Influences on Variability.

[21] Arkhipkin A.I., Rodhouse P.G.K., Pierce G.J., Sauer W., Sakai M., Allcock L., Arguelles J., Bower J.R., Castillo G., Ceriola L. (2015). World Squid Fisheries. Rev. Fish. Sci. Aquac..

[22] Ning Y. (2020). Research on Fishing Vessel Behavior Recognition Methods Based on Deep Learning. Master’s Thesis.

[23] Zhang F., Huang S., Dong K., Zhu H., Wang J., Gao M., Sun Y., Zhang H. (2026). Feature extraction and analysis of operational state for squid-jigging vessels in the Southwest Atlantic high seas based on vessel position data. Mar. Fish..

[24] Lambert G.I., Jennings S., Hiddink J.G., Hintzen N.T., Hinz H., Kaiser M.J., Murray L.G. (2012). Implications of Using Alternative Methods of Vessel Monitoring System (VMS) Data Analysis to Describe Fishing Activities and Impacts. ICES J. Mar. Sci..

[25] Farchadi N., Welch H., Braun C.D., Allyn A.J., Bograd S.J., Brodie S., Hazen E.L., Kerney A., Lezama-Ochoa N., Mills K.E. (2024). Marine Heatwaves Redistribute Pelagic Fishing Fleets. Fish Fish..

[26] MacQueen J.B., Le Cam L.M., Neyman J. (1967). Some Methods for Classification and Analysis of Multivariate Observations. Proceedings of the Fifth Berkeley Symposium on Mathematical Statistics and Probability.

[27] Chen X., Tian S., Guan W. (2014). Variations of Oceanic Fronts and Their Influence on the Fishing Grounds of *Ommastrephes bartramii* in the Northwest Pacific. Acta Oceanol. Sin..

[28] Wang Z., Chen G., Han Y., Ma C., Lv M. (2021). Southwestern Atlantic Ocean Fronts Detected from Satellite-Derived SST and Chlorophyll. Remote Sens..

[29] Zhang Y., Yu W., Chen X., Zhou M., Zhang C. (2022). Evaluating the Impacts of Mesoscale Eddies on Abundance and Distribution of Neon Flying Squid in the Northwest Pacific Ocean. Front. Mar. Sci..

[30] Maunder M.N., Punt A.E. (2004). Standardizing Catch and Effort Data: A Review of Recent Approaches. Fish. Res..

[31] West R.M. (2022). Best Practice in Statistics: The Use of Log Transformation. Ann. Clin. Biochem..

[32] Dormann C.F., Elith J., Bacher S., Buchmann C., Carl G., Carré G., García Márquez J.R., Gruber B., Lafourcade B., Leitão P.J. (2013). Collinearity: A Review of Methods to Deal with It and a Simulation Study Evaluating Their Performance. Ecography.

[33] Zuur A.F., Ieno E.N., Elphick C.S. (2010). A Protocol for Data Exploration to Avoid Common Statistical Problems. Methods Ecol. Evol..

[34] O’Brien R.M. (2007). A Caution Regarding Rules of Thumb for Variance Inflation Factors. Qual. Quant..

[35] Shi Y., Yan L., Zhang S., Tang F., Yang S., Fan W., Han H., Dai Y. (2025). Revealing the Effects of Environmental and Spatio-Temporal Variables on Changes in Japanese Sardine (*Sardinops melanostictus*) High Abundance Fishing Grounds Based on Interpretable Machine Learning Approach. Front. Mar. Sci..

[36] Tan Y., Bai Y. (2025). Antarctic Krill Habitat Suitability Variation in the Southern Ocean over 20 Years. Front. Mar. Sci..

[37] Zhang M. (2026). Marine Fishery Resource Dynamic Prediction Based on CNN-XGBoost Fusion Model. Sci. Rep..

[38] Wang L., Song L., Sui H., Li B. (2025). Interpretable Fish Abundance Index Prediction in Tuna Longline Fisheries: A LightGBM-SHAP Case Study in the Tropical Atlantic Ocean. Fish. Res..

[39] Pereira A.P., Anderson A.B., Gaurisas D.Y., Bernardino A.F. (2026). Effects of the Amazon River Plume in Benthic Distribution across the Amazon-Guianas Shelf. Front. Mar. Sci..

[40] Kim M.-J., Kim C., Kim H.W., Ji H.-S., Kang H. (2025). Forecasting the Spatial Variation of Optimal Sea Surface Temperature for Common Squid (*Todarodes pacificus*) in the Korean Jigging Fishery. Front. Mar. Sci..

[41] Liu Y., Zhang X., Zhou Y., Sun M. (2012). An analysis of biological characteristics of Argentine shortfin squid *Illex argentinus* collected by trawl in the Southwest Atlantic in 2011. South China Fish. Sci..

[42] Wang X., Wu J., Lin Y. (2023). Current status and development trends of fishery management for *Illex argentinus* in waters of the Argentine Exclusive Economic Zone. Fish. Inf. Strategy.

[43] Tang F., Cui X., Fan W., Zhang S., Zhang X., Wu Y. (2016). Study on relationship between resources abundance of squids and marine environment in high seas fishing grounds. J. Agric. Sci. Technol..

[44] Wu Y., Yang S., Shen J., Zhou W., Zhang J. (2009). Fishing grounds characteristics of *Illex argentinus* in Southwest Atlantic. Chin. J. Appl. Ecol..

[45] Fang Z., Chen X., Li J., Lu H. (2013). Distribution of fishing grounds of *Illex argentinus* and its relationship with sea surface temperature in waters of the Exclusive Economic Zone of Argentina. J. Shanghai Ocean Univ..

[46] De Boyer Montégut C., Madec G., Fischer A.S., Lazar A., Iudicone D. (2004). Mixed Layer Depth over the Global Ocean: An Examination of Profile Data and a Profile-Based Climatology. J. Geophys. Res. Oceans.

[47] Liu H., Yu W. (2025). Modeling the Potential Distribution of Argentine Shortfin Squid in the Southwest Atlantic Ocean. J. Oceanol. Limnol..

[48] Xiang D., Sun Y., Zhu H., Wang J., Huang S., Han H., Zhang S., Shang C., Zhang H. (2024). Prediction of the Relative Resource Abundance of the Argentine Shortfin Squid *Illex Argentinus* in the High Sea in the Southwest Atlantic Based on a Deep Learning Model. Animals.

[49] Li N., Lu H., Chen X. (2017). Comparison of different forecasting models for fishing grounds of *Illex argentinus* based on artificial neural networks in the Southwest Atlantic Ocean. J. Guangdong Ocean Univ..

[50] Wang J., Gao F., Lei L., Guan W., Chen X. (2014). Study on the forecasting model of *Illex argentinus* stock recruitment based on environmental factors in the spawning grounds. Haiyang Xuebao.

[51] Ding Q., Chen X., Wang J. (2015). Comparison of habitat suitability index models for *Illex argentinus* and their application to fishing-ground forecasting. Prog. Fish. Sci..

